# On the Maxillofacial Development of Mice, *Mus musculus*

**DOI:** 10.1002/jmor.70032

**Published:** 2025-02-28

**Authors:** Hiroki Higashiyama, Shunya Kuroda, Akiyasu Iwase, Naoki Irie, Hiroki Kurihara

**Affiliations:** ^1^ Research Center for Integrative Evolutionary Science (RCIES), The Graduate University for Advanced Studies, SOKENDAI Hayama Kanagawa Japan; ^2^ Department of Physiological Chemistry and Metabolism Graduate School of Medicine, The University of Tokyo Tokyo Bunkyo‐ku Japan; ^3^ Institute for Frontier Science Initiative Kanazawa University, Kakuma‐machi Kanazawa Ishikawa Japan; ^4^ Isotope Science Center, The University of Tokyo Tokyo Bunkyo‐ku Japan; ^5^ International Research Center for Medical Sciences (IRCMS) Kumamoto University Kumamoto Chuo‐ku Japan

**Keywords:** craniofacial, mammal, mouse, skull

## Abstract

The maxillofacial region is one of the most complex areas in the vertebrate body plan. The homology of the upper jaw bones remain controversial, both between mammals and nonmammalian amniotes and among humans and other mammals, leading to various hypotheses on how this region evolved from ancestral amniotes to humans. As a key mammalian model, the mouse (*Mus musculus*) is vital for unraveling the evolution and development of the maxillofacial region experimentally. However, limited detailed morphological descriptions of murine cranial development hinder the extrapolation of findings to other species, including humans. Here, we describe the development of the murine face, including the nerves, skeletons, and vasculatures from the pharyngula (9.0 days post‐coitum [dpc]) to the late fetal period (18.5 dpc) based on three‐dimensional reconstructions of histological sections. The present results confirm that the morphology of the pharyngula stages and developmental process of chondrocranium of mice is highly conserved when compared to nonmammalian tetrapods and humans. We also propose that the Os incisivum, the rostralmost bone in the mammalian upper jaw, consists of septomaxillary and palatine components, supporting our previous hypothesis that the ancestral premaxilla was entirely lost in mammals. The present descriptive study of mice strengthen the anatomical correspondence between mouse and human faces and offers a solid framework for comparative craniofacial studies across vertebrates.

## Graphical abstract

This study focused on the craniofacial development of the mouse (*Mus musculus*), particularly the maxillofacial region. Using three‐dimensional reconstructions of histological sections combined with whole‐mount staining, we provided a detailed description of skeletal, neural, and vascular formation from the pharyngula (9.0 dpc) to late fetal stages (18.5 dpc). Our data confirmed that the cranial base development in mice represents a typical mammalian pattern. Observations of the *Os incisivum* suggested that it comprises septomaxillary and palatine components, supporting the hypothesis that the ancestral premaxilla was lost in mammals. We also discussed human‐mouse comparisons, proposing an anatomically consistent framework for upper jaw evolution.

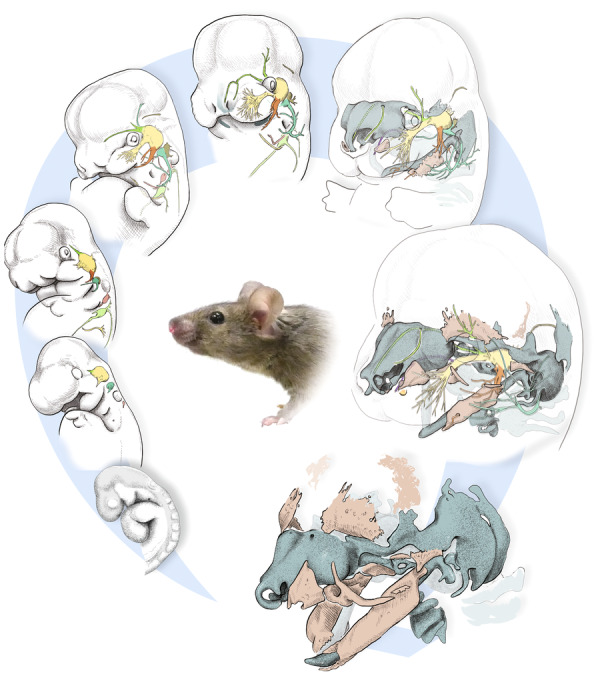

## 1 Introduction

In the early 1900s, C57BL/6 strain mice were first established by Clarence Cook Little from the common house mice bred by Abbie Lathrop (Steensma et al. 2010; Phifer‐Rixey and Nachman 2015). Since then, the mouse (*Mus musculus*) has been the most established mammalian model organism in the present biological‐ and medical sciences. Excellent textbooks have described the biology of mice in various approaches to date (Cook 1965; Theiler 1989; Kaufman 1992; Kaufman and Bard 1999; Rossant and Tam 2002; Ushiki et al. 2003; Baldock and Bard 2015). However, the morphological descriptions of mouse development are few, especially in the head region. The comparative morphologist Sir Gavin de Beer wrote in his famous book, *The Development of the Vertebrate Skull* (de Beer 1937), “no systematic study has been made of the development of the skull in either rats or mice, but from the figures given by Shindo (1915).” Indeed, several studies have described the cranial formation of mice using modern technology, such as genetic cell lineage tracing and micro‐CT scanning (McBratney‐Owen et al. 2008; Pitirri et al. 2020). But there is still no detailed description at a level comparable to the works of de Beer and Parker, which form the basis of the comparative morphology of the vertebrate head (e.g., Parker 1874, 1884a, 1884b; de Beer 1929, 1937; de Beer and Fell 1936).

The lack of detailed descriptions in mice prevents detailed comparisons between mice and nonmammalian model organisms or humans. Many homology issues remain unsolved in mammalian skulls (Koyabu 2023). One key issue is the longstanding debate regarding the homology of the premaxilla, the rostralmost upper jaw bone. Namely, it has long been debated whether the premaxilla is homologous across tetrapods or has been replaced by a nonhomologous bone in mammals (Broom 1895; Gaupp 1906; see Discussion for details). Recently, we reported that the premaxilla in mammals and nonmammalian tetrapods are not homologous bones, based on studies in mice (Higashiyama et al. 2021), although the homology of the chondrocranium, the basement of the craniofacial skeleton, remains underexplored. Even in comparisons between humans and other mammals, a consensus has not been reached regarding this region. While the human upper jaw, which appears to consist of paired maxillae in adults, clearly includes a premaxilla, if and how this structure is homologous to the premaxilla in mice and other mammals remains unclear (Reviewed by Barteczko and Jacob 2004). This challenge is particularly evident in cases of developmental abnormalities, such as bilateral cleft lip and palate (cleft lip/palate). In humans with cleft lip/palate, the so‐called premaxilla appears fully in the midline between the clefts, whereas in mice and many other mammals, the so‐called premaxilla typically forms laterally to the clefts, leaving only a small medial bony element (Biondi 1886; Inouye 1912; Bøhn 1963; Orr et al. 2016; Heyne et al. 2015). There have been few attempts to resolve such morphological inconsistencies using mice from a comparative morphological perspective. The classic morphological schemes of mice and men often diverge, especially in the maxillofacial region.

In the present study, we observed and described the development of the mouse head from the pharyngeal stage (9.0 dpc) to the fetal stage (18.5 dpc), with a focus on the maxillofacial region. Our goals are twofold: first, to provide a comprehensive morphological reference to communities using the mouse for a model organism, and second, to address the homology of the cranial base and upper jaw development to support comparisons with humans and nonmammalian tetrapods. Note that the rostralmost bone element of the mammalian upper jaw has often been referred to as the premaxilla, although its homology with the premaxilla of nonmammalian vertebrates has long been debated. Thus, we refer to this element of therian mammals as the *Os incisivum* (incisivum) throughout this paper, following Gaupp (1906) and several textbooks (Liem et al. 2000).

## 2 Materials and Methods

### 2.1 Laboratory Animal Specimens

Procedures involving animals were approved by the University of Tokyo Animal Care and Use Committee (Approval ID: P19‐043, P19‐050) and the guidelines of the Research Center for Integrative Evolutionary Science (RCIES), The Graduate University for Advanced Studies, SOKENDAI. We used a wild‐type mouse strain (C57BL/6J), which was kept in the laboratory at the University of Tokyo. The mice were housed in light‐ and dark‐cycled conditions (12 h light/12 h dark), temperature (25°C), humidity‐controlled conditions, and food and water were available ad libitum. Breeding pairs were housed together overnight, and the presence of a vaginal plug was used to confirm mating, which was assumed to occur at midnight and defined as 0 dpc. To obtain the embryos, we killed the pregnant mice as humanely as possible after being anesthetized, and cervical dislocation was performed. For the lineage tracing experiment of neural crest‐derived cells, *Wnt1‐Cre* mice (Jiang et al. 2000) were crossed with *Rosa26‐loxp‐stop‐loxp‐EYFP* (*R26‐EYFP*) reporter mice (Srinivas et al. 2001) to obtain *Wnt1‐Cre; R26‐EYFP* embryos.

### 2.2 Histological Sections

The embryos were fixed in modified Serra's fixative (4% PFA containing ethanol and acetic acid), dehydrated, and embedded in paraffin wax. Sections were cut at a thickness of 6–10 μm, depending on the size of the embryos. To visualize the nerve axons, we conducted immunohistochemistry. We used anti‐acetylated tubulin (Sigma‐Aldrich, no. T7451) as the primary antibody and HRP‐conjugated polyclonal goat anti‐mouse (Dako, no. P0447) as the secondary antibody. Subsequently, the sections were also stained with Alcian blue, hematoxylin, and eosin, following standard protocols.

### 2.3 Three‐Dimensional Imaging

For three‐dimensional reconstruction, the stained sections of the embryos were digitized using an Olympus BX60 microscope equipped with an Olympus DP70 camera and Olympus DP controller software (Olympus, Tokyo, Japan). The reconstruction was conducted by using the AMIRA 3D Visualization Framework (Thermo‐Fisher Scientific, Waltham, MA, USA).

### 2.4 Whole‐Mount Immunohistochemistry

To visualize the peripheral nerve in mice, we used monoclonal antibody 2H3 (Developmental Studies Hybridoma Bank, University of Iowa, Iowa City, IA, USA). The embryos were fixed with 4% PFA/PBS, washed and dehydrated in a graded series of methanol (70%, 95%), and stored at −30°C. Next, they were placed into a mixture of hydrogen peroxide and methanol (1:9) for several days for depigmentation and for blocking endogenous peroxidase activities. Then, 0.5 mL of 10% Triton X‐100 in distilled water was added, and the embryos were further incubated for 30 min at room temperature. After washing in Tris–HCl‐buffered saline solution (TST: 20 mmol L^−1^ Tris–HCl [pH 8.0], 150 mmol L^−1^ NaCl, 0.01% Triton X‐100), the samples were blocked with 5% nonfat dried milk in TST (TSTM). The embryos were then incubated in a primary antibody solution (diluted 1/100 in spin‐clarified TSTM containing 0.1% sodium azide) for 2–4 days at 37°C, while being gently agitated. The secondary antibody used was horseradish peroxidase (HRP)‐conjugated polyclonal goat anti‐mouse (Dako, Santa Clara, CA, no. P0447) diluted 1/400 in TSTM. After the final wash in TST, the embryos were incubated with peroxidase substrate 3,3′‐diaminobenzidine (100 μg mL^−^^1^) in TST with 0.01% (v/v) hydrogen peroxide (35% aqueous solution) for 20–40 min.

### 2.5 Skeletal Preparation

Skeletal staining of embryos was conducted using Alizarin red and Alcian blue solutions. Samples were fixed in 95% ethanol for 1 week, placed in acetone for 2 days, and then incubated with 0.015% Alcian blue 8GS, 0.005% Alizarin red S, and 5% acetic acid in 70% ethanol for 3 days. After washing in distilled water, the samples were cleaned in 1% potassium hydroxide for several days and in glycerol until the surrounding tissues turned transparent.

### 2.6 Drawings

**Figure 1:**
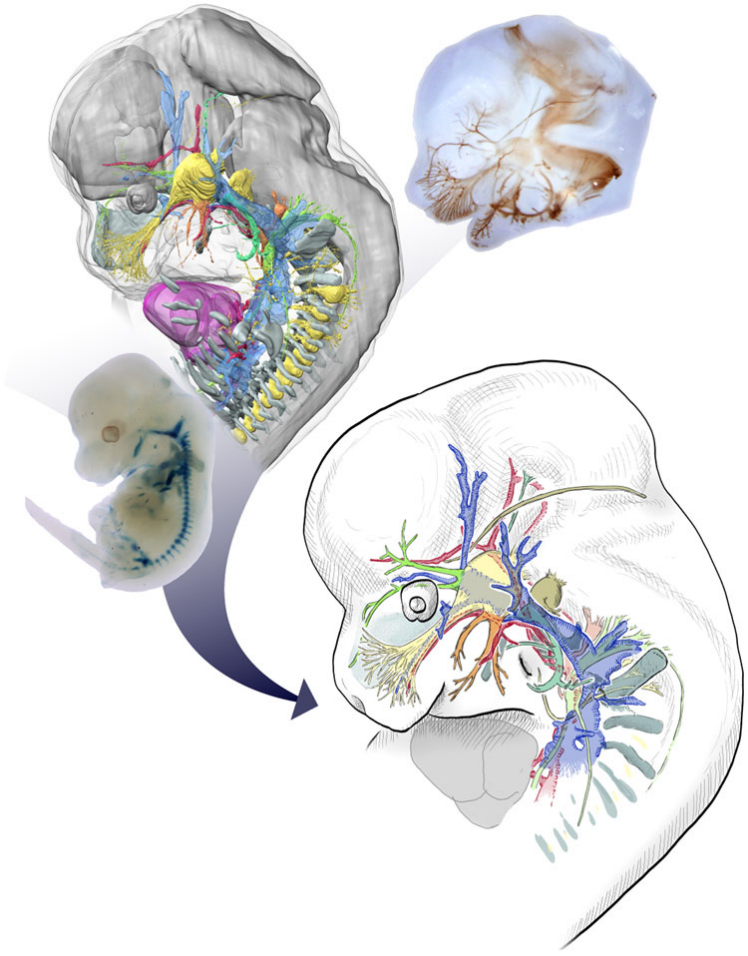
Observation and drawing. The samples are the 12.5 dpc mouse embryos. The figures in the main text are hand drawings. For the other data, see the Supporting Information.

In recent morphological studies, presenting results using photographs or three‐dimensional computer graphics have become common practice. However, these often fail to clearly convey the topographical relationships between structures, especially to readers without extensive background in morphology. This is particularly true for our three‐dimensional models, which are based on the histological sections and thus contain considerable distortion, making it difficult to understand the connections between the nerves and blood vessels (see the three‐dimensional images in the Supporting Information). Thus, following the approach of classical studies, we have created line drawings based on three‐dimensional models and whole‐mount stained samples (Figure 1). Note that some structures may differ slightly due to methodological or developmental differences. For example, the basicranial fenestra, which can be confirmed from tissue sections, is often unclear when whole‐mount stained with alizarin red and alcian blue (Figures 10 and S10). When such differences occur, the drawing is based on the three‐dimensional model.

The software used was Clip Studio Paint Pro (CELSYS Inc.), and the devices used were Wacom Cintiq 16 (Wacom Co. Ltd.) and iPad Pro (Apple Inc.). For the mouse skull at 18.5 days post coitum (dpc) in Figure 12, the drawing was done by hand on paper and scanned and digitally edited.

## 3 Results

### 3.1 9.0 dpc

**Figure 2:**
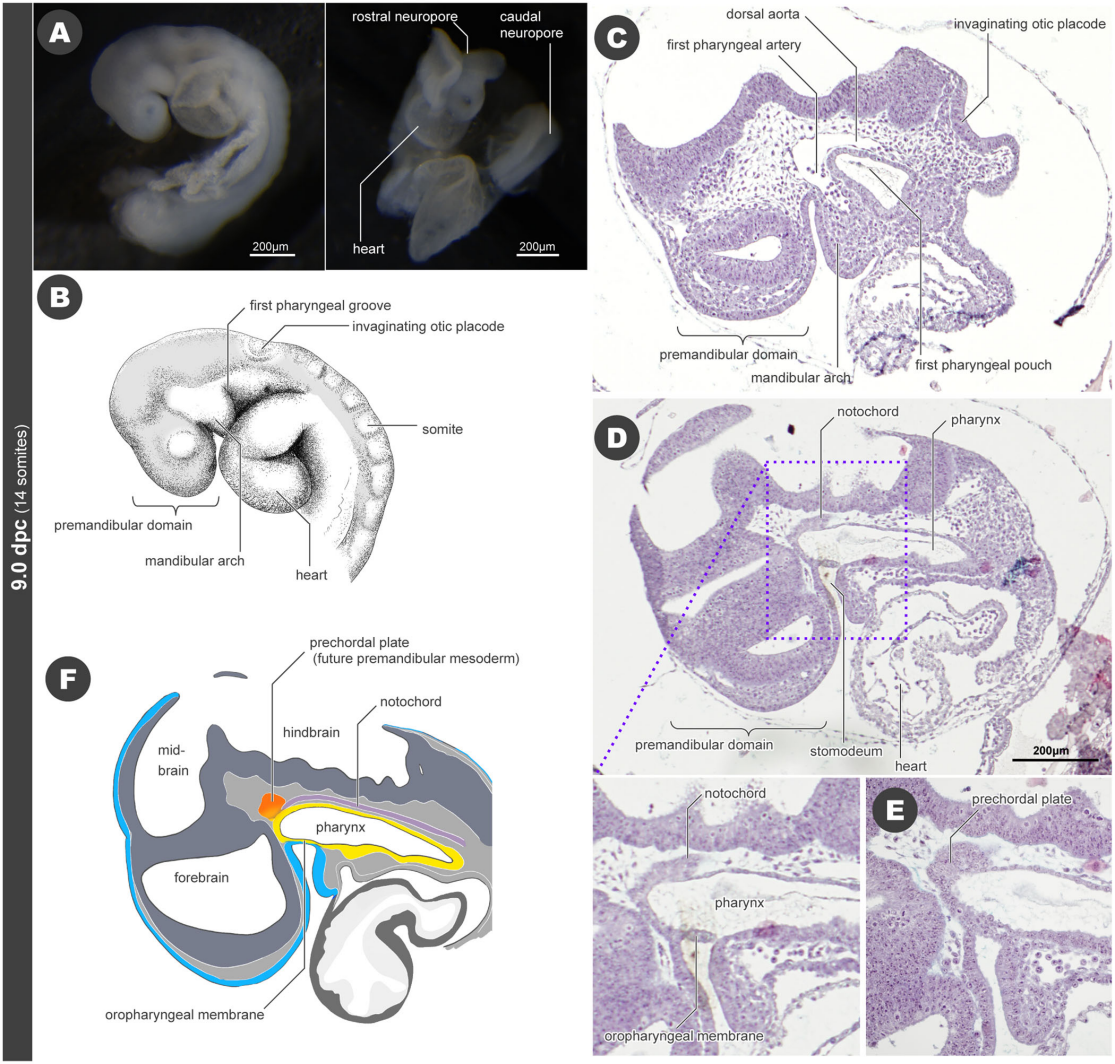
Morphology of mouse embryos at 9.0 dpc. (A) External appearance. The left panel shows the left lateral view and the right panel shows the front view. The neural tube has not yet closed completely, and the rostral and caudal parts are still open. (B) Sketch of external appearance. (C) Sagittal section of a 9.0 dpc embryo. (D) Sagittal section of a 9.0 dpc embryo. This is more medial than panel C. (E) The histological section, which is located more medial than panel D. (F) Scheme of the sagittal section at the midline, reconstructed from histological sections. The oral cavity, lined by ectoderm (blue), and the pharynx, lined by endoderm (yellow), are separated by the oropharyngeal membrane.

In 9.0 dpc embryos (14 somite‐stage), the neural tube is closed in the trunk region but remains open in both the cranial (rostral neuropore) and caudal regions (caudal neuropore) (Figure 2A). The mandibular arch (first pharyngeal arch) has already formed at this stage. The hyoid arch (second pharyngeal arch) is located caudal to the mandibular arch, separated by the first pharyngeal groove (Figure 2B). A ventral portion of the forebrain protrudes bilaterally to form the optic vesicles, which contact the lining of the ectodermal epidermis. The otic placode, forming the otic pit by invagination, is located dorsally at a slightly posterior level of the hyoid arch. The otic pit has not yet developed into an otic vesicle and remains bowl‐shaped and exposed to the outside of the body (Figure 2B,C). The heart is ventral to the developing pharynx (Figure 2A,B).

Histological sections of 9.0 dpc embryos show the three regions of the brain: forebrain (prosencephalon), midbrain (mesencephalon), and hindbrain (rhombencephalon). Rhombomeres are visible in the hindbrain in a segmental pattern (Figure 2C–E). A single pharyngeal artery (first pharyngeal artery) enters the mandibular arch from the dorsal aorta, connecting to the cardiac outflow tract; the pharyngeal artery of the hyoid arch (second pharyngeal artery) is still indistinct. Posterior to the mandibular arch, the endodermal pharyngeal epithelium overhangs to form the first pharyngeal pouch, which is in contact with the ectodermal pharyngeal groove on its lateral side but has not yet penetrated to form a pharyngeal slit (Figure 2C). At this developmental stage, the oropharyngeal membrane persists between the ectodermal lining of the stomodeum (oral cavity) and the endodermal lining of the pharynx. This membrane is laterally a multilayered epithelium, but closer to the midline it is a monolayer. Based on histological sections, it is challenging to determine which of the two germ layers this membrane originates from. The rostral end of the notochord touches the dorsal roof of the pharynx at a level slightly posterior to the oropharyngeal membrane. The prechordal plate is found in this location, which give rise to the premandibular mesoderm, a source of the future extraocular muscles (Figure 2D–F; see Grimaldi et al. 2022; Kuroda et al. 2023).

**Figure 3:**
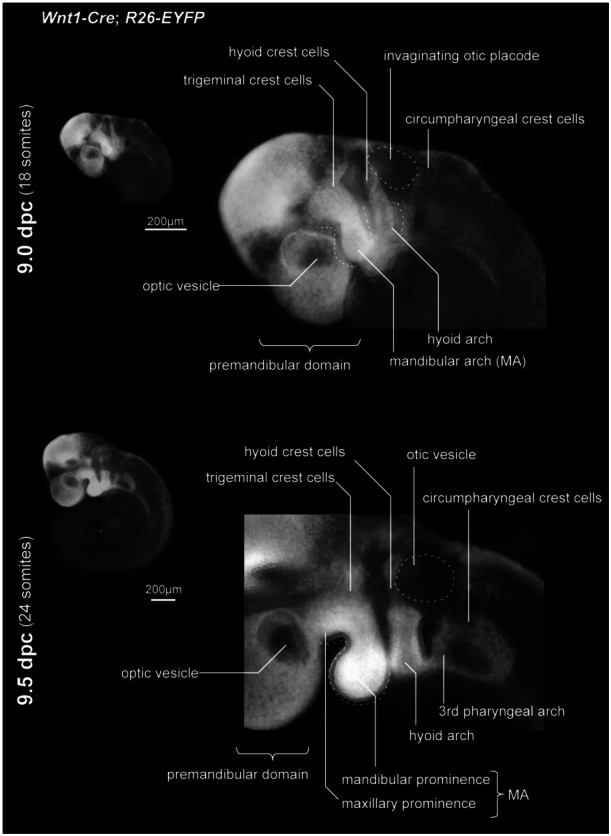
Distribution of cranial neural crest cells in 9.0 and 9.5 dpc embryos.

The *Wnt1‐Cre*; *R26‐EYFP* mice allow us to observe the three cranial neural crest cell populations, trigeminal‐, hyoid‐, and circumpharyngeal crest cells (Kuratani and Kirby 1991; Kuratani 1996) (Figure 3), which can be observed in other vertebrates (Noden 1991; Olsson and Hanken 1996; Falck et al. 2000; Kundrát 2009; Stundl et al. 2020). The rostralmost population, the trigeminal crest cells, migrate into the mandibular arch and the more anterior domain surrounding the forebrain, known as the premandibular domain. The hyoid crest cells migrate slightly rostral to the otic vesicle and occupy the hyoid arch. As the name implies, the circumpharyngeal crest cells, or post‐otic crest cells, distribute posterior to the otic placode and fill the third and more caudal pharyngeal arches. Some subpopulations of the circumpharyngeal crest cells are also called the vagal crest cells or cardiac crest cells, and as their names suggest, they are distributed in the vagus nerve's innervating area (e.g., heart and gastrointestinal tract) and the heart. Note that studies have shown that pre‐otic crest cells (trigeminal and hyoid crest cells) also contribute to the outflow tract of the heart, as well as the cardiopharyngeal mesoderm (Arima et al. 2012).

### 3.2 9.5 dpc

**Figure 4:**
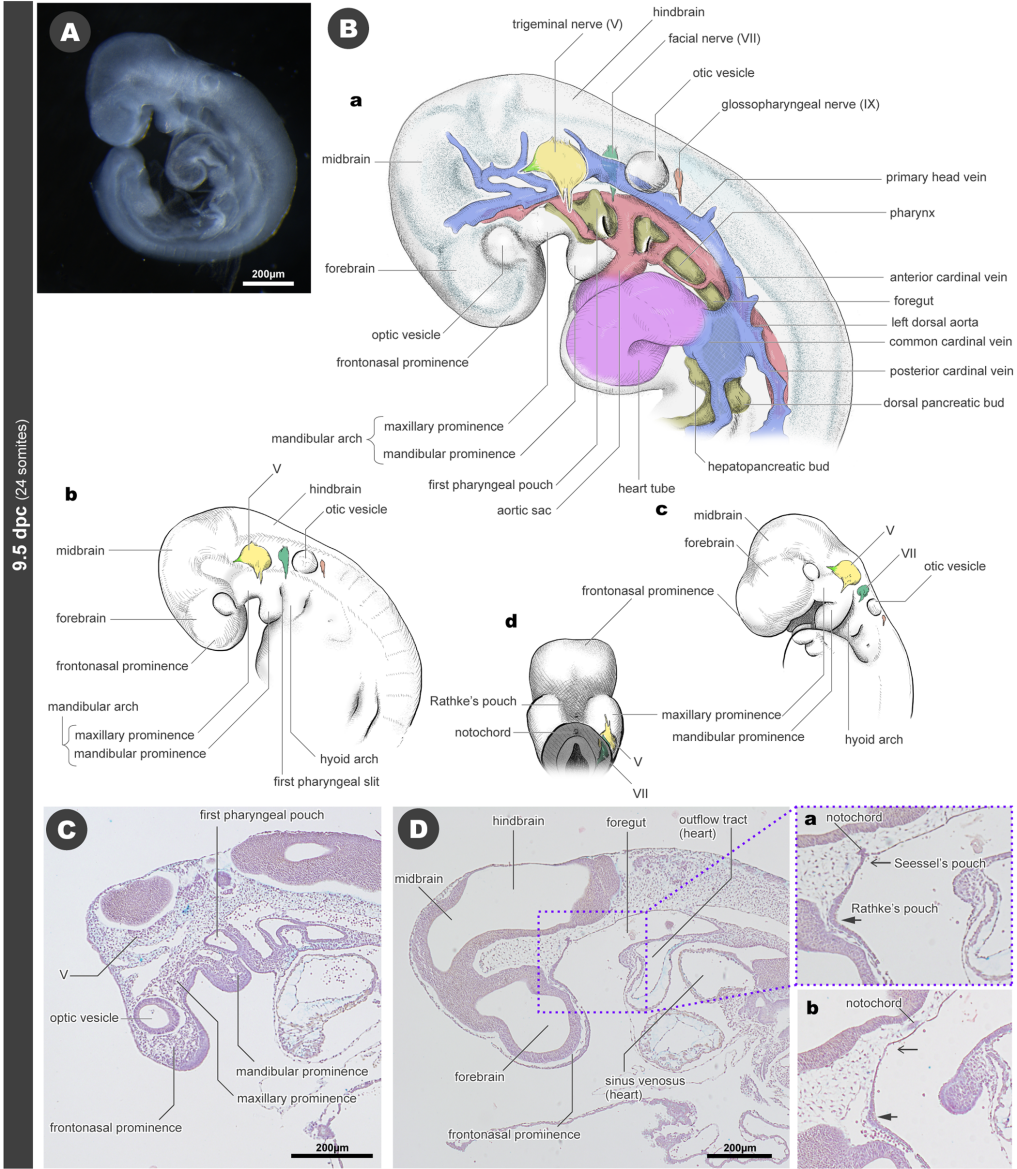
Morphology of mouse embryos at 9.5 dpc. (A) External appearance. (B) Drawings of the morphology. (a) Nerves, arteries, veins, and digestive tract in the head of the embryo. (b) Left lateral view. (c) Left diagonal view. (d) Palatal view. (C) Sagittal section. (D) Sagittal section. More medial than panel C. Panel‐a is an enlarged view of the area around the boundary between the oral cavity and pharynx. Panel‐b is a sagittal section around the boundary between the oral cavity and pharynx. The section is 12 µm to the right of panel Da.

In 9.5 dpc mice (24 somite‐stage), the neural tube is completely closed rostrally, while the caudal neuropore remains open (data not shown). At this developmental stage, the optic vesicle remains a simple vesicular form, and the lens has not yet been formed. The vesicular‐shaped otic vesicle forms, although a small portion still opens to the outside of the body as a　vestige of the otic pit condition. (Figure 4A,B).

The pillar‐shaped pharyngeal arches form around the pharynx (Figures 4A,B and S1). The pharyngeal grooves and pouches grow deeper and penetrate to form the pharyngeal slits (Figure 4Ba,b). In mice, two rostral grooves penetrate as slits throughout development. The third pharyngeal groove and pouch also form, but they do not penetrate as slits throughout their development (Figure 4Ba,C). Four pharyngeal arteries are on each side, connecting the dorsal aorta and the aortic sac (Figure 4B).

In the premandibular domain, the trigeminal crest cells fill the ventral region of the forebrain to form the rostralmost mesenchymal projection, termed the frontonasal prominence (Figure 4B–D). The mandibular arch appears to be bent dorsoventrally. The dorsal part can be identified as the maxillary prominence, while the ventral part becomes the mandibular prominence (Figure 4B–D).

The oropharyngeal membrane is lost at this stage, making the oral cavity and pharynx continuous. From the stomodeum, a pouch forms near the ventral floor of the forebrain, known as Rathke's pouch, which will become the adenohypophysis (Figure 4Da). The rostral end of the notochord still touches the dorsal roof of the pharynx, and a small pouch is found at this position, which should be termed Seessel's pouch (Figure 4Da,b). By comparing with the 9.0 dpc, the position of the oropharyngeal membrane (i.e., the ectoderm‐endoderm boundary) is estimated to be located between these two pouches.

### 3.3 10.5 dpc

**Figure 5:**
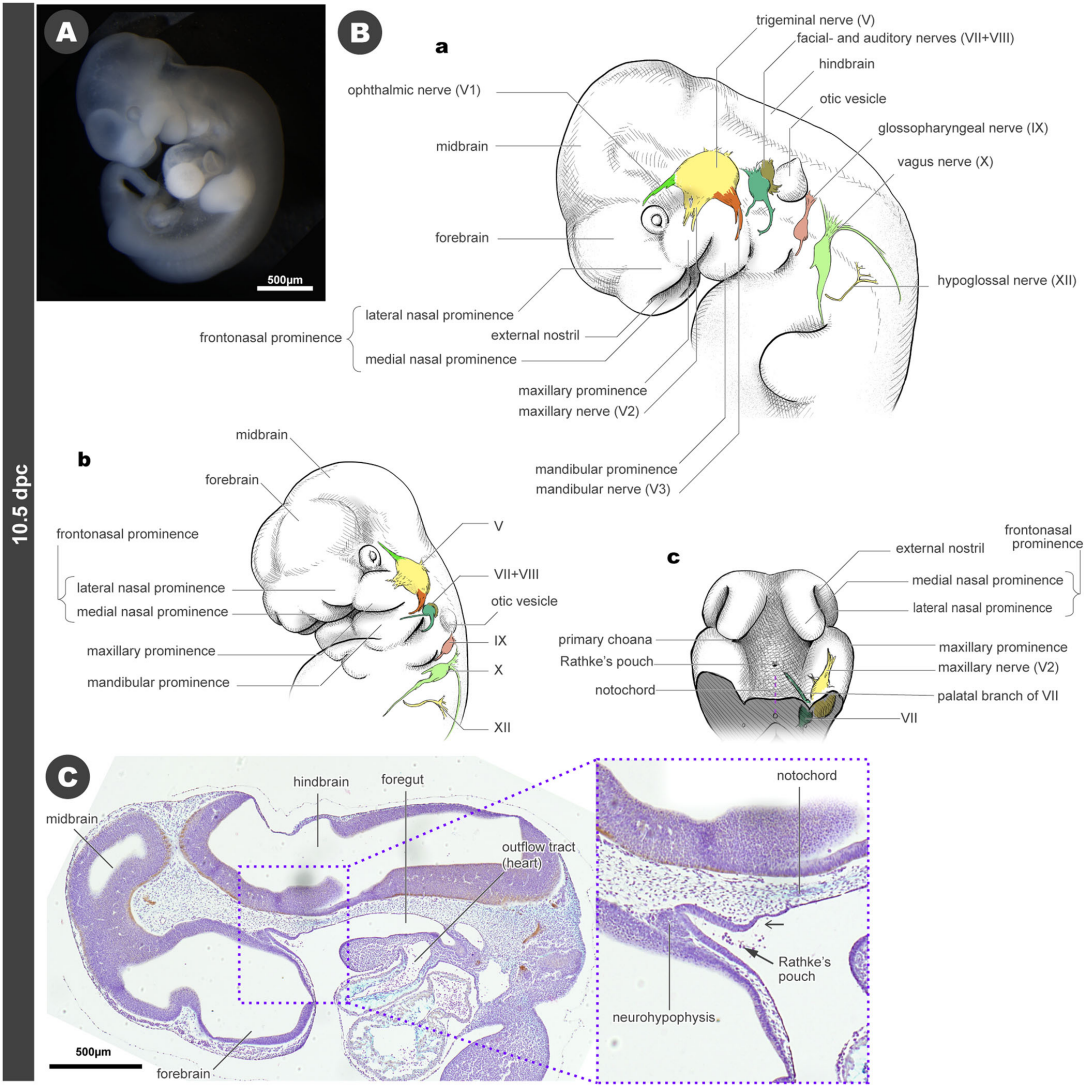
Morphology of mouse embryos at 10.5 dpc. (A) External appearance. (B) Drawings of the morphology. (a) Left lateral view. (b) Left diagonal view. (c) Palatal view. (C) Sagittal section. As with Figure 4Da, an arrow with a black triangular arrowhead indicates Rathke's pouch and a V‐shaped arrowhead indicates Seessel's pouch.

By 10.5 dpc, the optic vesicle becomes a cup‐shaped optic cup, and the primordial lens is derived from the adjacent epithelial placode. The otic pit closes, and thus, the internal space of the otic vesicle is completely separated from the outside of the embryo (Figure 5A; also see references).

At 10.5 dpc, the maxillary and mandibular prominences of the mandibular arch become more distinctly. The hyoid arch is also recognized as an independent structure. However, posterior to the third pharyngeal arch, no distinct arch shape can be identified from the external view (Figure 5A,B). The nasal pits (future external nostrils) form in the premandibular domain, separating the frontonasal prominence medio‐laterally. The medial projections between the developing external nostrils are defined as the medial nasal prominences, while the lateral ones are called lateral nasal prominences (Figure 5B). A profound frontal groove separates the medial nasal prominences in the midline (Figure 5Bb,c).

At this developmental stage, the cranial nerves become histologically visible. The trigeminal nerve has three branches: the ophthalmic (V1), maxillary (V2), and mandibular nerves (V3), which supply the premandibular domain (passing dorsal to the optic nerve [II]), and the maxillary and mandibular prominences, respectively (Figures 5Ba, S1, and S7). Additionally, the facial nerve (VII), auditory nerve (VIII), glossopharyngeal nerve (IX), and vagus nerve (X) are formed, each accompanied by their respective ganglia at 10.5 dpc. Note, however, that the VII and VIII ganglia are almost always attached to each other, making it difficult to separate them by immunohistochemical staining. The hypoglossal nerves (XII) also become visible as a set of branches of the rostralmost spinal nerves; thus, the position of XII indicates the head‐trunk mesenchymal interface (Kuratani 1996). At 10.5 dpc, XII is located just lateral to the common cardinal vein (ductus Cuvieri) connecting to the sinus venosus, a constituent of the cardiac inflow tract, while this relationship shifts as the neck elongates in the subsequent ontogenetic processes. (Figures 5Ba and 13A; also see Higashiyama et al. 2016).

Rathke's pouch is still open at the roof of the oral cavity (Figure 5Bc,C). The pouch is adjacent to the ventromedial part of the forebrain, and the primordium of the neurohypophysis differentiates from this part of the brain (Figure 5C). Immediately posterior to Rathke's pouch, a subtle depression is present, presumed to be Seessel's pouch. The rostral end of the notochord still makes contact with this subtle pouch. It is not known whether the boundary between ectoderm and endoderm is robustly maintained through this developmental stage. Although the pouch is generally regarded as the ectodermal derivative, in zebrafish, a small number of endodermal cells contribute to Rathke's pouch‐derived pituitary gland (Fabian et al. 2020). Whether the same occurs in mice or not remains uncertain.

### 3.4 11.5 dpc

**Figure 6:**
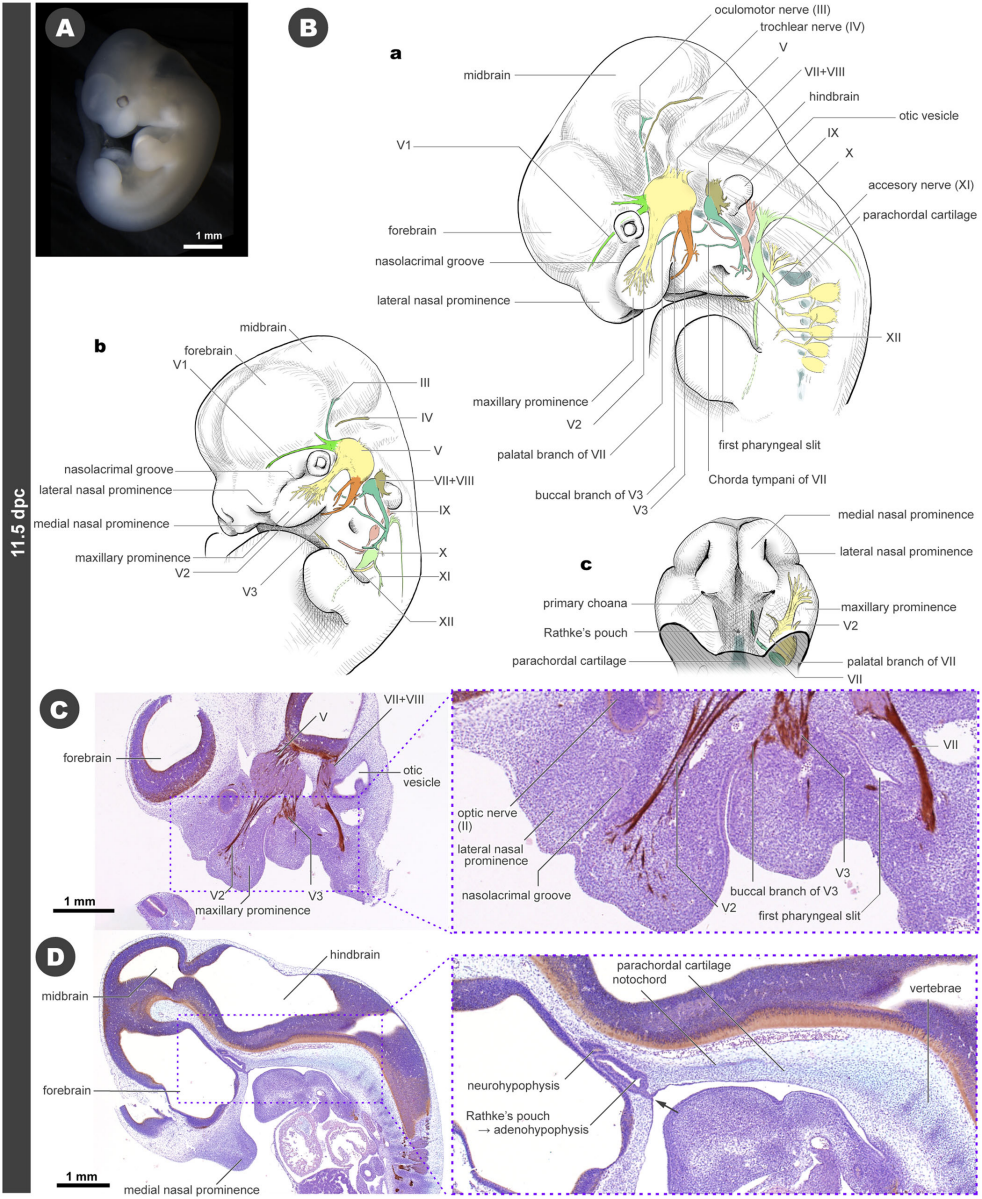
Morphology of mouse embryos at 11.5 dpc. (A) External appearance. (B) Drawings of the morphology. (a) Left lateral view. (b) Left diagonal view. (c) Palatal view. (C) Sagittal section at the level of the maxillary prominence. (D) Sagittal section at the midline.

At 11.5 dpc, the facial prominences are still distinguishable, although they begin to fuse (Figure 6A). The frontal groove becomes more constricted, facilitating the merging of the medial nasal prominences. Simultaneously, the maxillary prominences exhibit rostral growth, with their rostralmost portions attaching laterally to the medial nasal prominences. The nasolacrimal groove is located between the lateral nasal and maxillary prominences (Figure 6Bb,C). The left and right mandibular prominences fuse at the midline, contributing to the formation of the lower jaw. The hyoid and posterior pharyngeal arches also undergo fusion, obscuring the boundaries of the arches, except for the first pharyngeal slit (future ear canal) (Figure 6Ba,c).

The peripheral distribution of the cranial nerves also increases in complexity (Figure 6B). The ophthalmic (V1) nerve courses dorsal to the optic nerve (not shown in the figure) and extends in the frontal region, ventrolateral to the forebrain. The maxillary nerve (V2) has small peripheral branches, most of which are destined to become the infraoptic nerve for the maxillary vibrissae, extends within the maxillary prominence. The mandibular nerve (V3) exhibits several branches. The buccal branch from the major trunk of the V3 nerve supplies the sensation to angle of the mouth. Apart from the buccal branch, the major branches of V3 extends in an arcuate pattern to the lower jaw (Figures 6Ba, C, and S7).

The major trunk of the facial nerve (VII) curves in an arc towards the posterior aspect of the first pharyngeal slit (Figure 6Ba,b,C). The chorda tympani branches from the major trunk and extends rostrally on the dorsal side of the first pharyngeal slit, eventually ending near V3 (Figure 6Bb). At later developmental stages, the chorda tympani joins the lingual nerve of V3, which provides sensory innervation to the tongue and oral cavity (Standring 2005; Schünke et al. 2006). The palatine nerve, originating directly from the facial ganglion, extends rostrally over the dorsal surface of the pharynx to reach the primary roof of the oral cavity (Figures 6Ba,c and S7). At this developmental stage, several small branches arise from the root of the V2 nerve and pass medially through the maxillary prominences to the primary oral roof. Some of these branches make anastomoses with the palatine nerve (Figure 6Bc).

At the same developmental stage (11.5 dpc), the glossopharyngeal (IX) and vagus (X) nerves undergo enlargement (Figure 6B). The accessory nerve (XI) shares the same root as the X nerve (for details on the accessory nerve, see Tada and Kuratani 2015). The hypoglossal (XII) nerve forms an arc and extends rostrally in the median plane, with its proximal end directed toward the future tongue primordium. The X nerve extends fine branches to the pharynx, with some separating from the body wall slightly rostral to the XII nerve arc and distributing across the gastrointestinal tract as intestinal rami (for details, see Higashiyama et al. 2016).

At this developmental stage, sagittal sections reveal a substantial increase in the mesenchyme of the rostromedial part of the face, resulting from the merging of the left and right medial nasal prominences at the midline (Figure 6D). The opening of Rathke's pouch to the oral cavity becomes closed, forming an independent structure known as the adenohypophysis. It attaches to the neurohypophysis from the ventral aspect of the forebrain. The Seessel's pouch could not be identified in this stage. The discernible traces of the notochord remain rudimentary. Posterior to the adenohypophysis, we observed the cellular condensation of the parachordal cartilage, the caudal part of the cranial base, and the rudimentary notochord (Figure 6D). These aggregates extend in a manner contiguous with the segmented vertebrae of the trunk.

### 3.5 12.5 dpc

**Figure 7:**
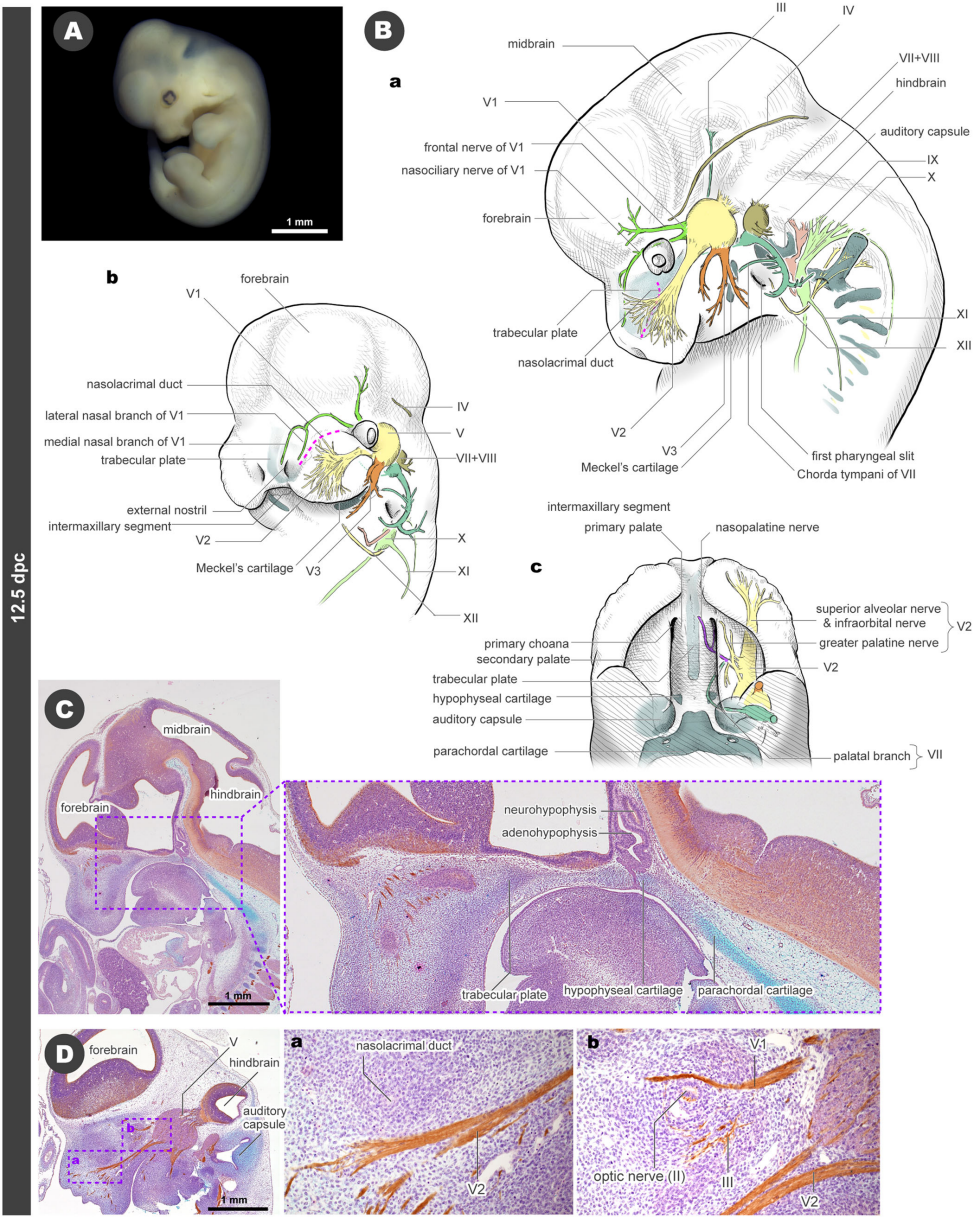
Morphology of mouse embryos at 12.5 dpc. (A) External appearance. (B) Drawings of the morphology. (a) Left lateral view. (b) Left diagonal view. (c) Palatal view. (C) Sagittal section at the midline. (D) Sagittal section at the level of the maxillary prominence. The panels a and b are the magnified images from panel D.

At 12.5 dpc, the facial prominences fuse with each other, forming the upper and lower lips (Figure 7A). The boundaries of the primordia become unclear, even histologically, and can only be identified by some external reliefs. At this developmental stage, the pair of medial nasal prominences merge at the midline, forming the narrow intermaxillary segment between the maxillary prominences (Figure 7Bb,c). A sagittal section reveals the condensation for the chondrocranium in the mid‐facial region, forming an unpaired trabecular plate (Figure 7B,C). This prechordal skeleton is faint at 12.5 dpc, making it difficult to observe in whole‐mount skeletal staining (Supporting Information, Figure S9). Posterior to the trabecular plate, the hypophyseal cartilage arises separately, surrounding the adenohypophysis (see Figure 7Bc,C). In contrast, the parachordal cartilage becomes solid and is easily stained by Alcian blue (Figure 7C). The otic vesicle is also surrounded by cartilage called the auditory capsule or otic capsule (Figure 7Bc,D). In the lower jaw, Meckel's cartilage first appears.

The maxillary prominence covers most of the upper jaw, and the observable boundaries with other prominences are limited to the shallow groove bordering the intermaxillary segment (Figure 7Bb). On the palatal side of the maxillary prominences, the secondary palates extend like eaves toward the midline, beginning to cover the primary palatal roof from the ventrolateral sides (Figure 7Bc). The boundary between the maxillary and lateral nasal prominences becomes indistinguishable externally, causing the nasolacrimal groove to disappear. Only the nasolacrimal duct, which connects the eye and nasal cavity, remains as a remnant of the nasolacrimal groove (Figures 7B,D, and S3). The diameter of this duct is as small as a few cells, but it can be identified through hematoxylin–eosin staining, and its histological continuity can be followed from eye to nasal cavity (Figure 7Da).

Some characteristic branches appear in the cranial nerves. The V1 nerve gives rise to two major branches, the frontal and nasociliary nerves, caudal to the eye (Figures 7Ba, S3, and S8). Posterior to the nasal cavity, the nasociliary nerve divides into two major branches: the medial nasal branch and the lateral nasal branch (Figure 7Bb). The former extends in the medial nasal prominence region along the dorsal part of the nasal septum bilaterally, while the latter encircles the future nasal capsule from the lateral sides. These V1 nerve branches never reach the oral ridge, even in the intermaxillary segment.

The bundle of the infraorbital and the anterior alveolar branches of V2 is visible from the lateral view (Figures 7Ba, S3, and S8). These branches separate just posterior to the eye in 12.5 dpc mice, although the alveolar nerve extends in close proximity to the infraorbital nerve, making them appear as a single nerve bundle. From the palatal view, several branches of the V2 nerve extend toward the palate and anastomose with the palatine branch of the VII nerve. From this anastomosis (the future pterygopalatine ganglion), the greater palatine nerve extends toward the secondary palate, and the nasopalatine nerve extends toward the primary palate (Figures 7Bc and S8). The nasopalatine nerve passes ventral to the trabecular plate and medial to the two primary choanae (Figures 7Bc and S8).

### 3.6 13.5 dpc

**Figure 8:**
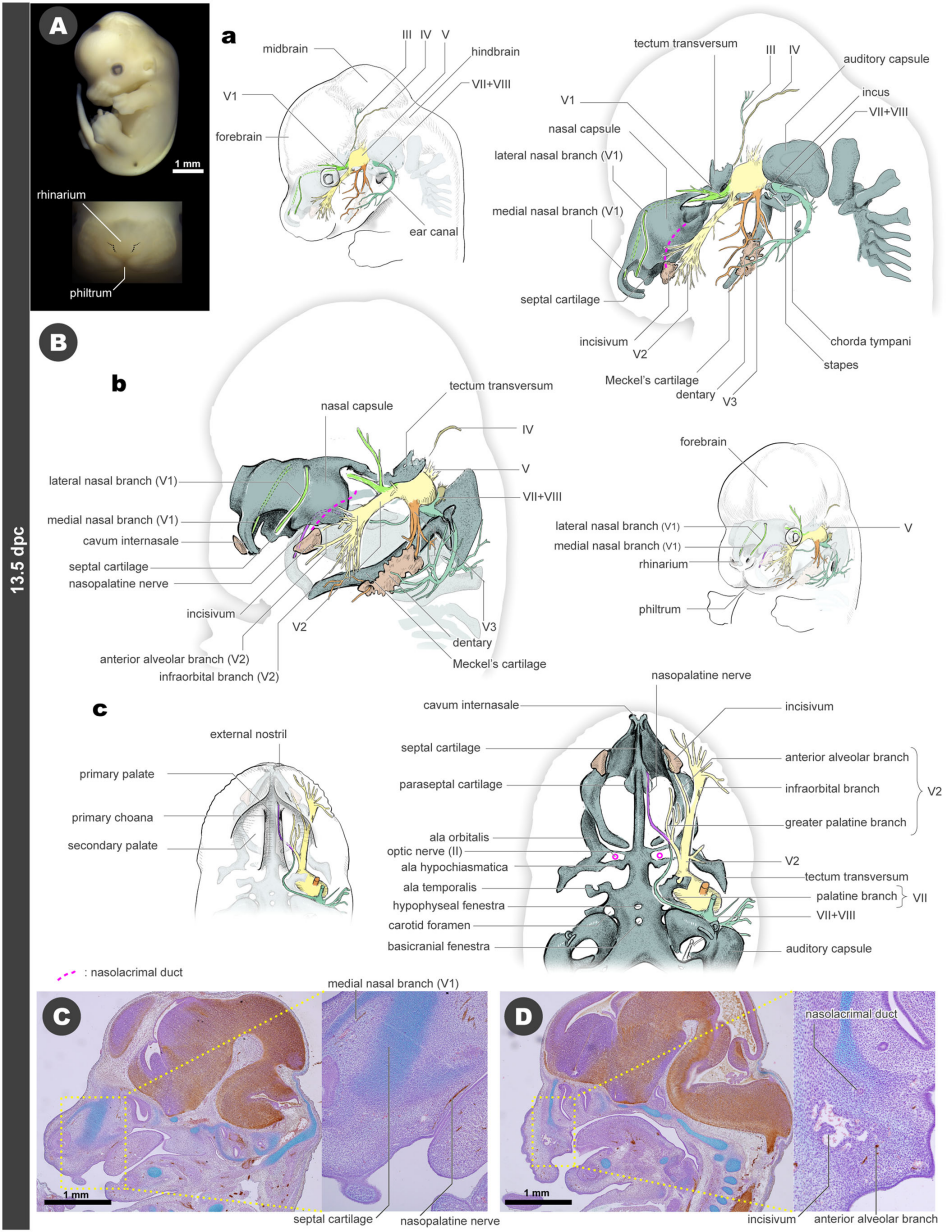
Morphology of mouse embryos at 13.5 dpc. (A) External appearance. The lower panel is the frontal view, showing the philtrum. (B) Drawings of the morphology. (a) Left lateral views. (b) Left diagonal views. (c) Palatal views. (C) Sagittal section at the almost midline. (D) Sagittal section at the level of the incisivum.

The maxillary prominences, with the primordia of hair follicles of vibrissae, enlarge to cover the entire upper jaw as the upper lip. These prominences envelop the ventrolateral layer of the intermaxillary segment and fuse at the facial midline, forming the philtrum—a distinctive feature of mammalian faces. This arrangement of facial primordia causes the medial nasal prominence to separate from the oral ridge, establishing the protruding nose at the center of the face, a unique trait of mammals (Figure 8A; see also Higashiyama et al. 2021).

At this stage, the trabecular plate becomes a solid cartilage connected to the hypophyseal and parachordal cartilages, forming the cranial base (Figure 8B,C). Rostral to the trabecular plate, the septal cartilage extends to form the skeleton of the nasal septum, which vertically supports the snout in a sagittal plane. Ventral to the septal cartilage, paired paraseptal cartilages arise and cover the vomeronasal organs ventrally (Figures 8Bc and S10). The nasal capsule also forms, enclosing the nasal cavity. On the rostral side of the narial fenestra (the nasal opening of the chondrocranium), the cartilage of the nasal capsule forms a small domed roof called the cupola nasi anterior (Figure S10). Between these two cupolas lies a shallow V‐shaped valley known as the cavum internasale, where the rostral end of the septal cartilage also terminates (Figures 8Bb,c and S10). This cavum internasale is located behind the rhinarium, with the cartilages around this area forming the nasal cartilage during later developmental stages.

The nasal septum and capsule are associated with the distribution of the V1 nerve. The frontal nerve of V1 extends from just behind the eye to the frontal region, while the nasociliary nerve extends through the nasal capsule. The left and right medial nasal branches pass through the roof of the nasal capsule along both sides of the nasal septum and reach the rostral end of the neurocranium, reaching into the cavum internasale (Figure 8Ba,b,C). The lateral nasal branch passes through the foramen epitheliale to exit the nasal capsule and is distributed on the surface of the snout toward the rostral side (Figure 8Ba,b). As seen in the 12.5 dpc embryos, the nasopalatine nerve is found on the cranial base. It emerges from the pterygopalatine anastomosis, enters the nasal septum, passes ventrally along the septal cartilage, and emerges at the epidermis of the primary palate, between the primary choana (Figure 8Bb,c,C). Thus, the nasal region of the mouse is primarily innervated by the V1 nerves, with nasopalatine nerves specifically supplying the ventral midline.

On either side of the nasal capsule, the incisivum, i.e., the rostralmost bone of the murine upper jaw, ossifies just posterior to the narial fenestra of the nasal capsule. The ossification center is located besides the rostral opening of the nasolacrimal duct at 13.5 dpc and lateral to the anterior alveolar branch of the V2 nerve (Figures 8B,D, and S4). The infraorbital branch, the largest branch of the V2 nerve, covers the upper jaw structures and gives rise to fine branches to the upper lip and vibrissae (Figure 8B). The greater palatine branch of the V2 nerve is distributed in the secondary palate, as observed at 12.5 dpc (Figure 8Bc).

The left and right Meckel's cartilage extend rostrally and the distal ends meet at the midline of the lower jaw. Additionally, hinge‐shaped joints (primary jaw joints) are formed at the proximal caudal end, which articulate with the newly formed incus (Figure 8Ba,b). The incus attaches to the stapes, which is positioned in the fenestra ovalis of the auditory capsule.

In the lower jaw, the dentary ossifies lateral to Meckel's cartilage, covering the inferior alveolar branch of the V3 nerve (Figure 8Ba,b). The VII nerve's main branches extend across the lateral aspect of the lower jaw, likely corresponding to the development of the facial musculature. The primordium of the buccinator muscle can be recognized on the lateral surface near the VII nerve (Figure S4).

The adenohypophysis becomes separated from the oral cavity, and the notochord disappears. However, the hypophyseal fenestra is still histologically observable in the chondrocranial base, which should correspond to the rostral end of the area where the notochord was originally located (Figure 8Bc). Although the ala hypochiasmatica, located just behind the optic nerve (II), is continuous with the trabecular cranii in the three‐dimensional reconstruction of this study, it appears as an independent cartilaginous condensation in the whole‐mount skeletal staining (Figures 8Bc and S10). This independent development may indicate distinct developmental origins for the ala hypochiasmatica and the trabecular cranii cells (McBratney‐Owen et al. 2008).

### 3.7 14.5 dpc

**Figure 9:**
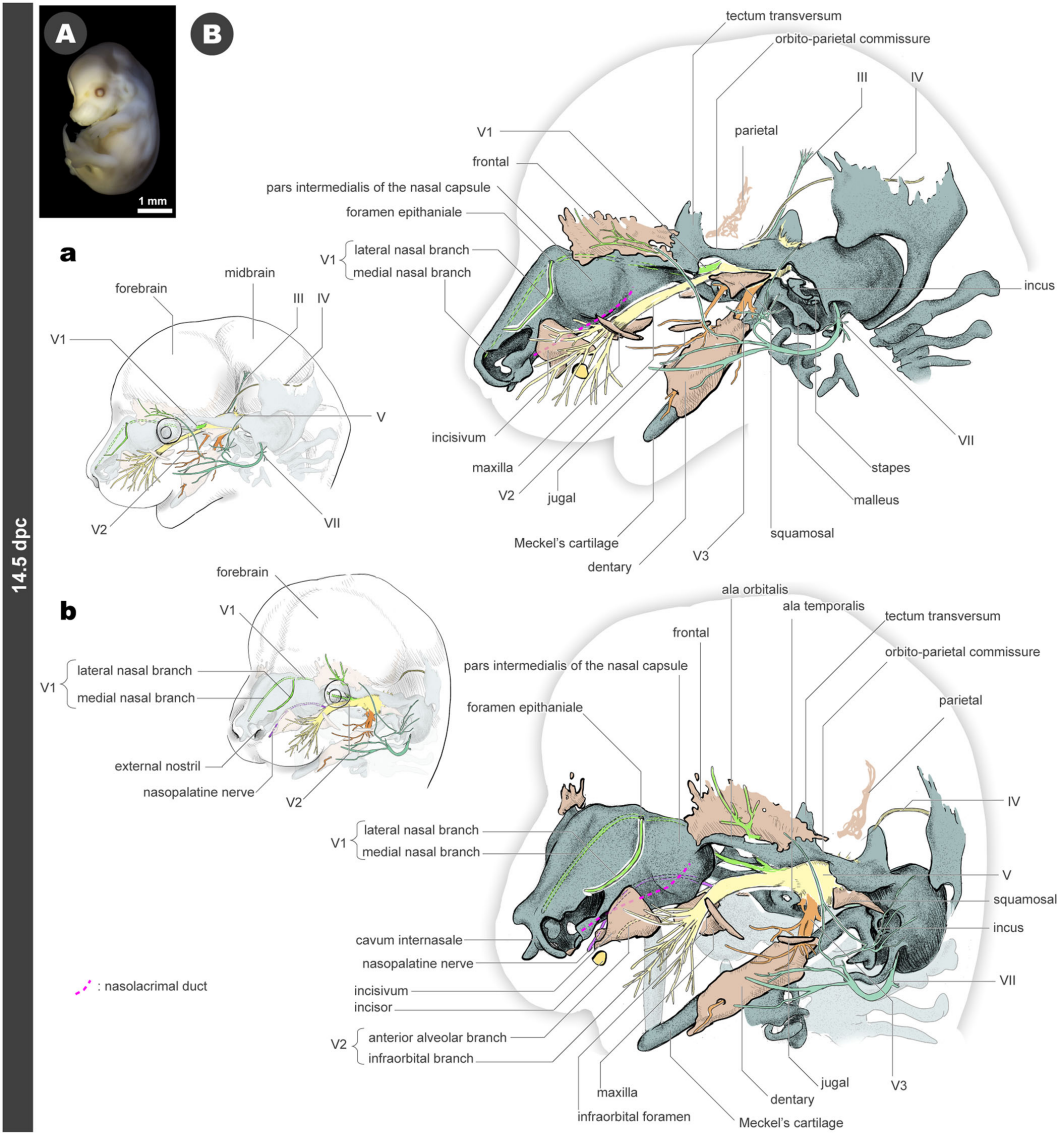
Morphology of mouse embryos at 14.5 dpc. (A) External appearance. (B) Drawings of the morphology. (a) Left lateral views. (b) Left diagonal views.

The chondrocranium exhibits a complex three‐dimensional architecture. Similar to 13.5 dpc, the medial nasal branches of the V1 nerve extend along the ceiling of the nasal cartilage on both sides of the septal cartilage, reaching the rostral tip of the nose (Figure 9Ba,C). Caudal to the tectum transversum, a cartilage structure forms an arch called the orbito‐parietal commissure, which laterally covers the trigeminal ganglion (Figure 9B). The pila preoptica is found anterior to the optic nerve, creating an optic foramen bounded by this structure and the ala hypochiasmatica (Figure 9Bc). The hyoid apparatus also emerges, with the styloid process derived from the hyoid arch and the hyoid bone appearing as cartilage (Figures 9Ba and 10A). The major branches of the VII nerve extend parallel to the styloid process (Figures 9Ba and 10A).

At this developmental stage, the incisivum undergoes further ossification. As at 13.5 dpc, the anterior alveolar branch of the V2 nerve penetrates this bone (Figures 9Bb and 10A). The rostral end of the nasolacrimal duct shifts anteriorly compared to the 13.5 dpc embryos, passing medially to the incisivum and opening just behind the narial fenestra. Histologically, the primordium of the incisor tooth is observable close to the incisivum, although it remains in the oral epithelium and is not yet in contact with the ossified incisivum.

**Figure 10:**
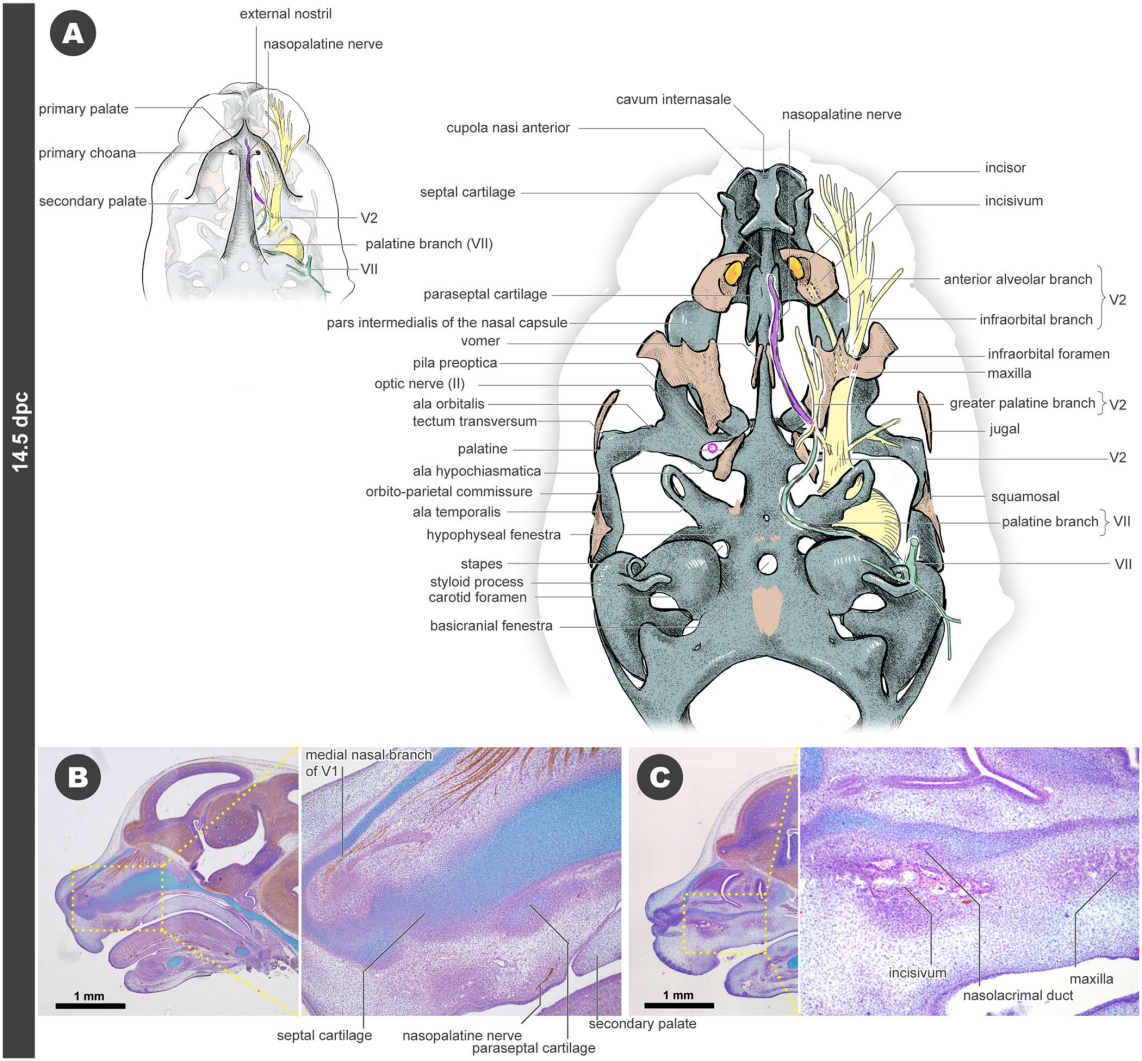
Morphology of mouse embryos at 14.5 dpc (continue). (A) Drawings of the morphology. Palatal views. (B) Sagittal section at the almost midline. (C) Sagittal section at the level of the incisivum.

Posterior to the incisivum, the maxilla forms ventrolaterally to the pars intermedialis of the nasal capsule (Figures 9, 10, and S10). From a ventral perspective, this bone initiates the formation of the infraorbital foramen, covering the V2 nerve branches, specifically the anterior alveolar and infraorbital branches.

In addition to the jaw bones, other bony elements also ossify at this stage. The jugal appears at the cheek to form the zygomatic arch (Figures 9, 10, and S5). The squamosal begins ossifying on the outer side of the orbito‐parietal commissure (Figures 9, 10, and S5). From the palate side, a pair of vomers are seen ventral to the septal cartilage and caudal to the paraseptal cartilage (Figures 9, 10, and S10). Beneath the ala hypochiasmatica, the palatine bone also begins to ossify (Figures 10A and S10). The ossifications of the frontal and parietal bones initiate the formation of the cranial roof (Figures 9, 10, and S5).

The trigeminal nerve (V) has become more finely branched, although the topographical positions of the major branches have not significantly changed since 13.5 dpc. The V1 nerve is distributed to the frontal and nasal capsule, while the V2 nerve covers most of the upper jaw (Figures 9, 10, and S5). The nasopalatine nerve passes ventral to the septal cartilage, near the ossification site of the vomer, and supplies innervation around the primary palate, ventral to the paraseptal cartilage (Figure 10A, B). At 14.5 dpc, the peripheral branches of the VII nerve are widely distributed on the facial surface (Figures 9Ba, b, and S5), which is related to the development of facial musculature, such as the buccinator muscle (Figure S5).

### 3.8 15.5 dpc

**Figure 11:**
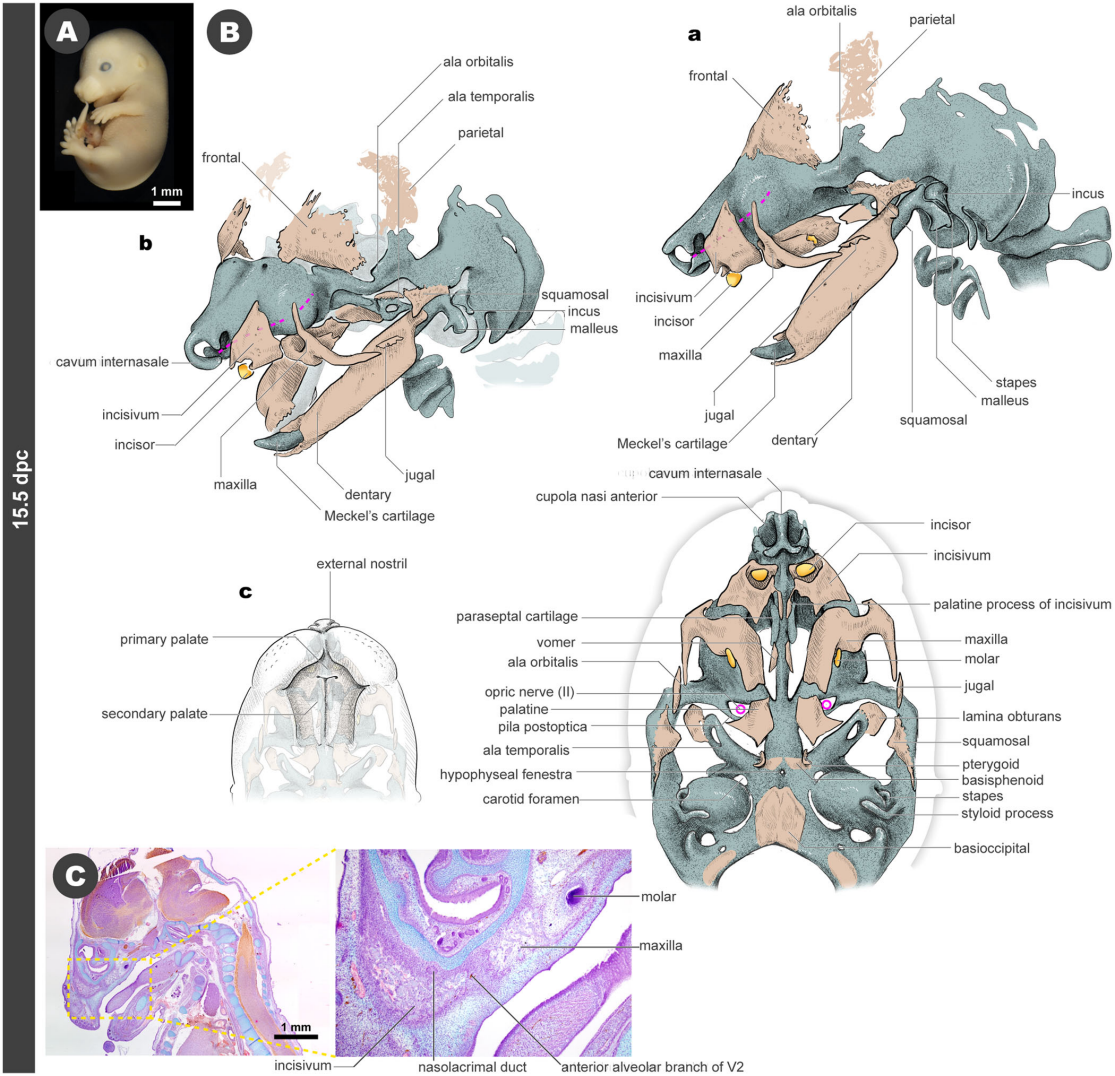
Morphology of mouse embryos at 15.5 dpc. (A) External appearance. (B) Drawings of the morphology. (a) Left lateral view. (b) Left diagonal view. (c) Palatal views. (C) Sagittal section at the level of the incisivum.

At 15.5 dpc (Figure 11A), nearly all the morphological features characteristic of mice are present. The secondary palate fuses at the midline, while the nasopalatine canal persists between the primary palate and the secondary palatal area (Figure 11Bc).

The incisivum continues to grow, forming the dental socket for the incisor. The nasolacrimal duct passes medially to the incisivum, opening beside the narial fenestra (Figure 11B). From a palatal view, the palatine processes of the incisivum are first seen at the position of the primary palate, ventral to the septal cartilage, covering the rostral half of the paraseptal cartilages (Figure 11Bc). During the normal development of mice, the palatine process does not appear to separate from the lateral body at any developmental stages. The maxilla undergoes ossification, enclosing the molar teeth (Figure 11Bc,C). The anterior alveolar branch of the V2 nerve enters the maxilla, sends branches to the molars, then passes through the suture between the incisivum and the maxilla, and subsequently distributes branches to the incisor after entering the incisivum (Figure 11C).

The dentary of the lower jaw covers almost the entire length of Meckel's cartilage from the lateral aspect, with its caudal end forming a secondary jaw joint with the squamosal of the upper jaw. At this developmental stage, the caudal end of Meckel's cartilage (which will eventually develop into the malleus) and the incus still form the primary jaw joint, resulting in the transient coexistence of dual (ancestral and derived) jaw joints (Figures 11Ba,b and S6).

### 3.9 18.5 dpc

**Figure 12:**
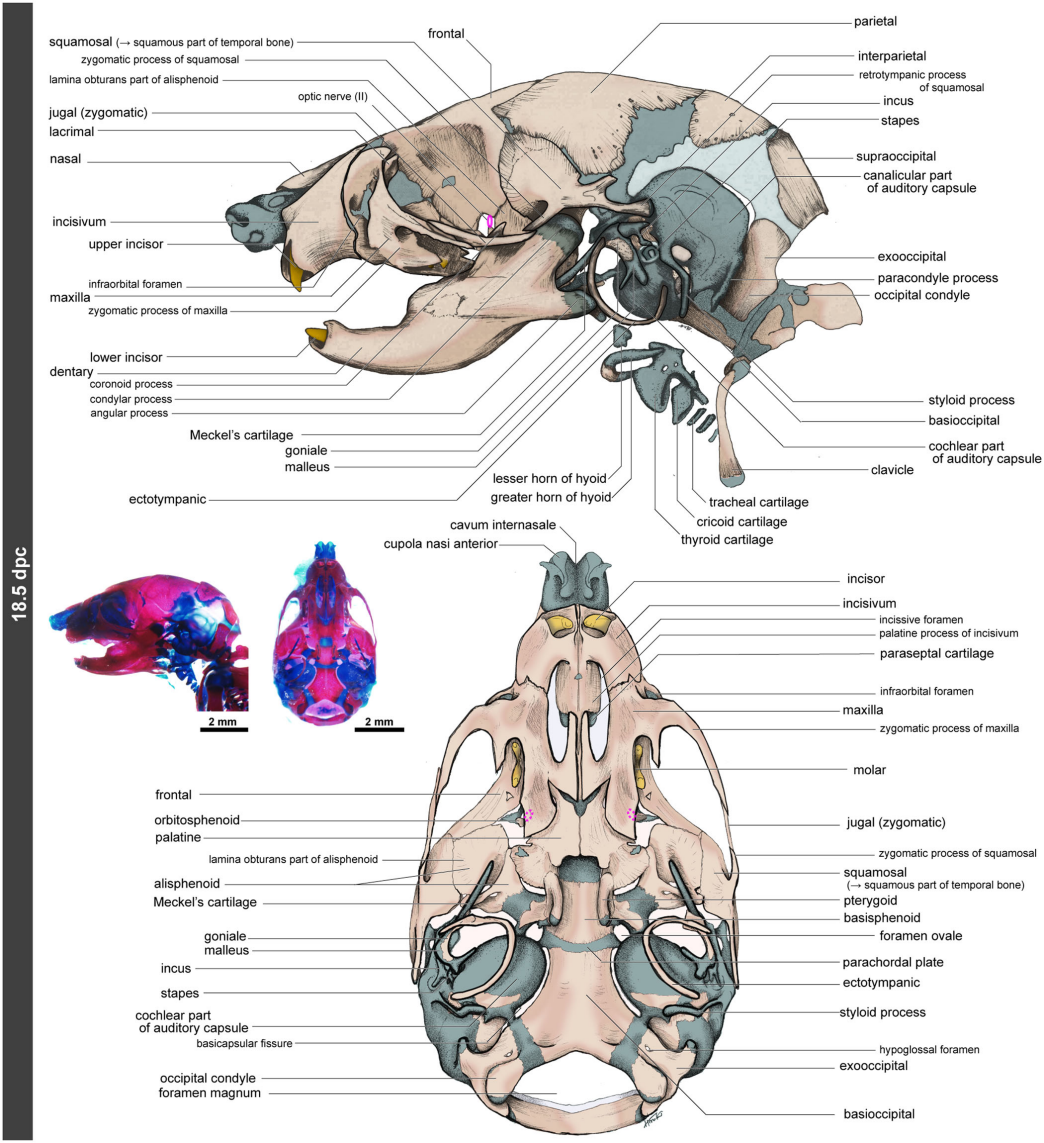
Morphology of mouse embryos at 18.5 dpc. The pink oval is the position of the optic nerve (it passes through the optic foramen). From the palatal side, it is covered by the palatine bones, so we cannot see it (pink dotted oval).

At 18.5 dpc, just before birth, the head morphology of the mouse is almost indistinguishable from that of newborn pups (Figures 12 and S11). The nasal bone ossifies, covering the nasal cartilage from the dorsal side. The left and right incisivum bones meet at the midline. The palatine process has developed, covering most of the paraseptal cartilage from the ventral aspect. The vomer is positioned just behind the palatine process of the incisivum, although it cannot be observed externally due to the palatine process of the maxilla covering its palatal side. The maxilla, jugal, and squamosal bones connect through the zygomatic processes of each bone, forming the zygomatic arch. The secondary jaw joint further differentiates, with the condylar process exhibiting well‐developed articular cartilage. At 18.5 dpc, the malleus is barely separated from the remaining part of Meckel's cartilage, and at this stage, the mouse still possesses two jaw joints. The goniale begins to ossify on the surface of the malleus, and ring‐shaped ectotympanic forms around the middle ear.

### 3.10 Arteries and Veins

The distribution of blood vessels has traditionally provided key landmarks for comparative morphology across species, although arteries and veins frequently undergo anastomosis, branching, and pathway changes more often than skeletal and neural structures, making their developmental tracing challenging. In this section, we focus on the progression of major arteries and veins in addition to the skeletal and neural observations.

At 9.5 dpc, arteries and veins are positioned internally and externally along the body axis. Four distinct pharyngeal (arch) arteries were observed, extending from the cardiac outflow tract to the dorsal aorta located dorsally to the pharynx (Figures 4Ba and S1). The caudal (inflow) region of the heart is connected to the common cardinal vein (ductus Cuvieri), which in turn receives the anterior cardinal vein and the posterior cardinal vein (Figures 4Ba and S1). Thus, the vascular system at this developmental stage closely reflects the basic plan of the vascular system described by de Beer (1937).

**Figure 13:**
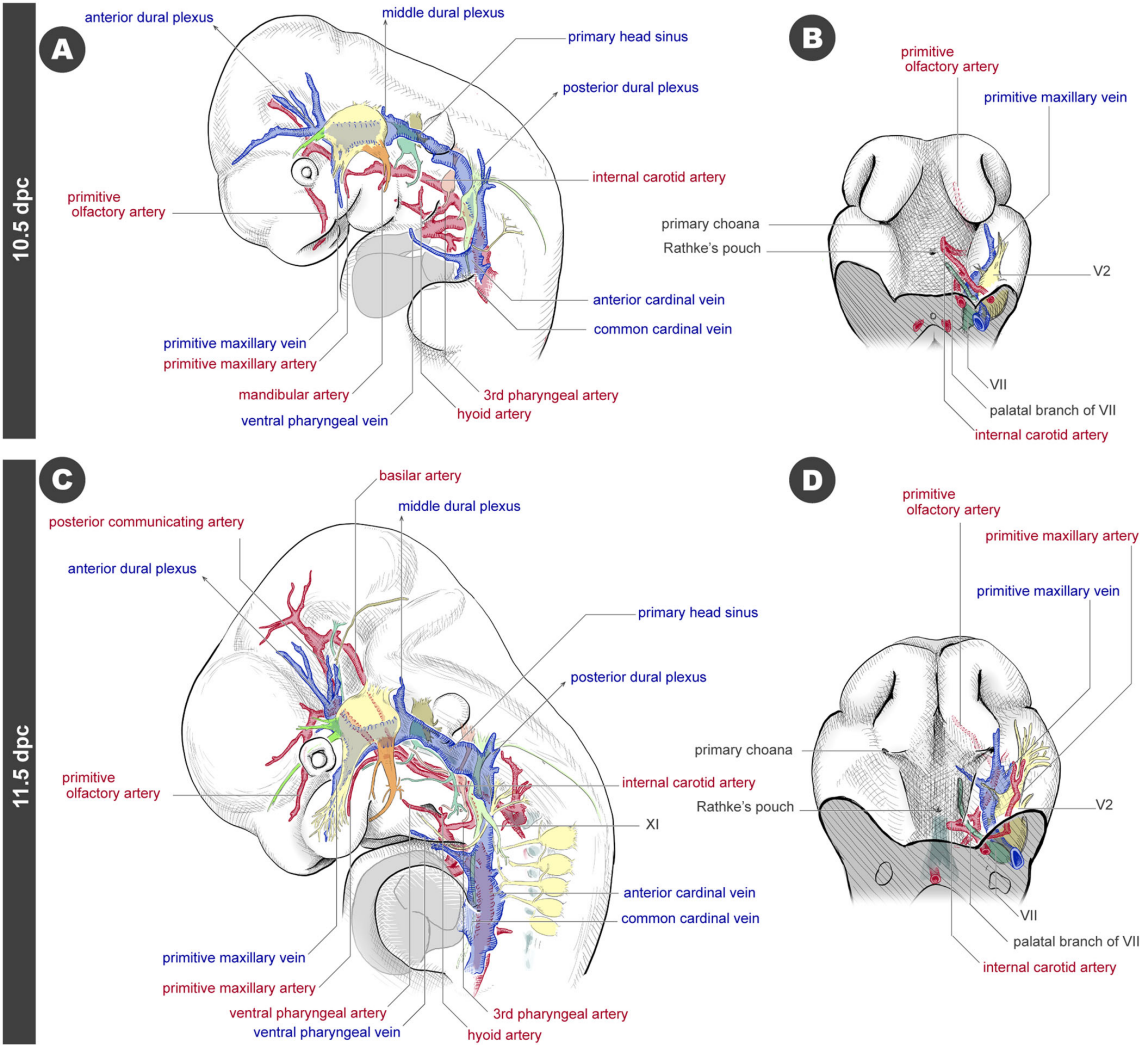
Arteries and veins at 10.5 and 11.5 dpc embryos. (A) Left lateral view of the 10.5 dpc embryo. (B) Palatal view of the 10.5 dpc embryo. (C) Left lateral view of the 11.5 dpc embryo. (D) Palatal view of the 11.5 dpc embryo.

By 10.5 dpc, coinciding with the differentiation of the pharyngeal arches, the anterior two pharyngeal arteries—the mandibular artery and the hyoid artery—regress (Figures 13A and S1). At this stage, the portion of the dorsal aorta rostral to the original branching point of the hyoid artery is referred to as the internal carotid artery. When viewed from the palatal side, the internal carotid artery curves medially, passing close to Rathke's pouch (Figure 13B). Additionally, the primitive maxillary artery becomes visible within the maxillary prominence. Regarding venous development, three dural plexuses—anterior, middle, and posterior—form anterior to the trigeminal ganglion, posterior to the trigeminal ganglion, and posterior to the vagus nerve, respectively (Figures 13A and S1).

At 11.5 dpc, the basilar artery, which extends along the base of the brain (pons and medulla) in adults, becomes prominent (Figures 13C and S2). It connects to the internal carotid artery via the posterior communicating artery. The mandibular artery is no longer observable, and the hyoid artery has further regressed. The internal carotid artery continues to pass close to Rathke's pouch, as it did at 10.5 dpc (Figure 13D). On the venous side, the primary head sinus, located medial to the vagus ganglion at 10.5 dpc, has shifted laterally relative to the vagus nerve, likely due to vascular remodeling around the posterior dural plexus (Figures 13B and S2). The XI nerve, which branches from the vagus nerve trunk, is now distributed laterally to the primary head sinus.

**Figure 14:**
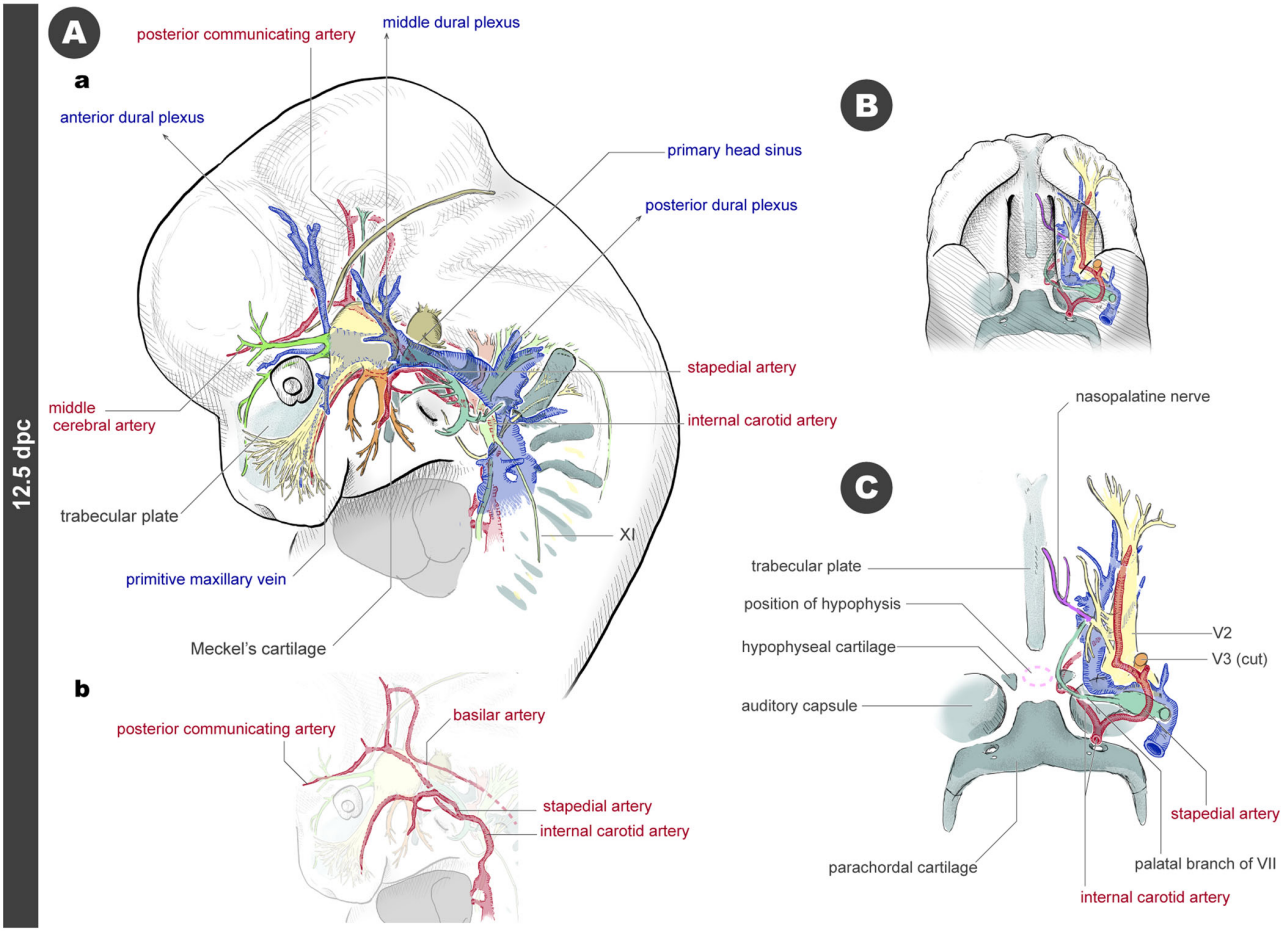
Arteries and veins at 12.5 dpc embryos. (A) Left lateral views. Panel a shows arteries, veins, and nerves. Panel b shows only the course of arteries. (B) Palatal view. (C) Palatal view. The outline lines are removed from Panel B.

By 12.5 dpc, the arteries form a large loop when viewed laterally, extending from the internal carotid artery to the basilar artery (Figures 14A and S3). The remnant of the regressed hyoid artery has now transformed into the stapedial artery. From a ventral view, the internal carotid artery branches from the stapedial artery and curves medially just before entering the cranial base, passing near the derivative of Rathke's pouch, which has now differentiated into the adenohypophysis (Figure 14B,C). The hypophyseal cartilages form on either side of the hypophysis at this developmental stage. The internal carotid artery passes caudal to the hypophyseal cartilages, passing dorsally to the cartilages before curving laterally. The dural plexuses continue to develop in the venous system, with the middle dural plexus now covering the posterolateral side of the trigeminal ganglion (Figure 14A and S3).

**Figure 15:**
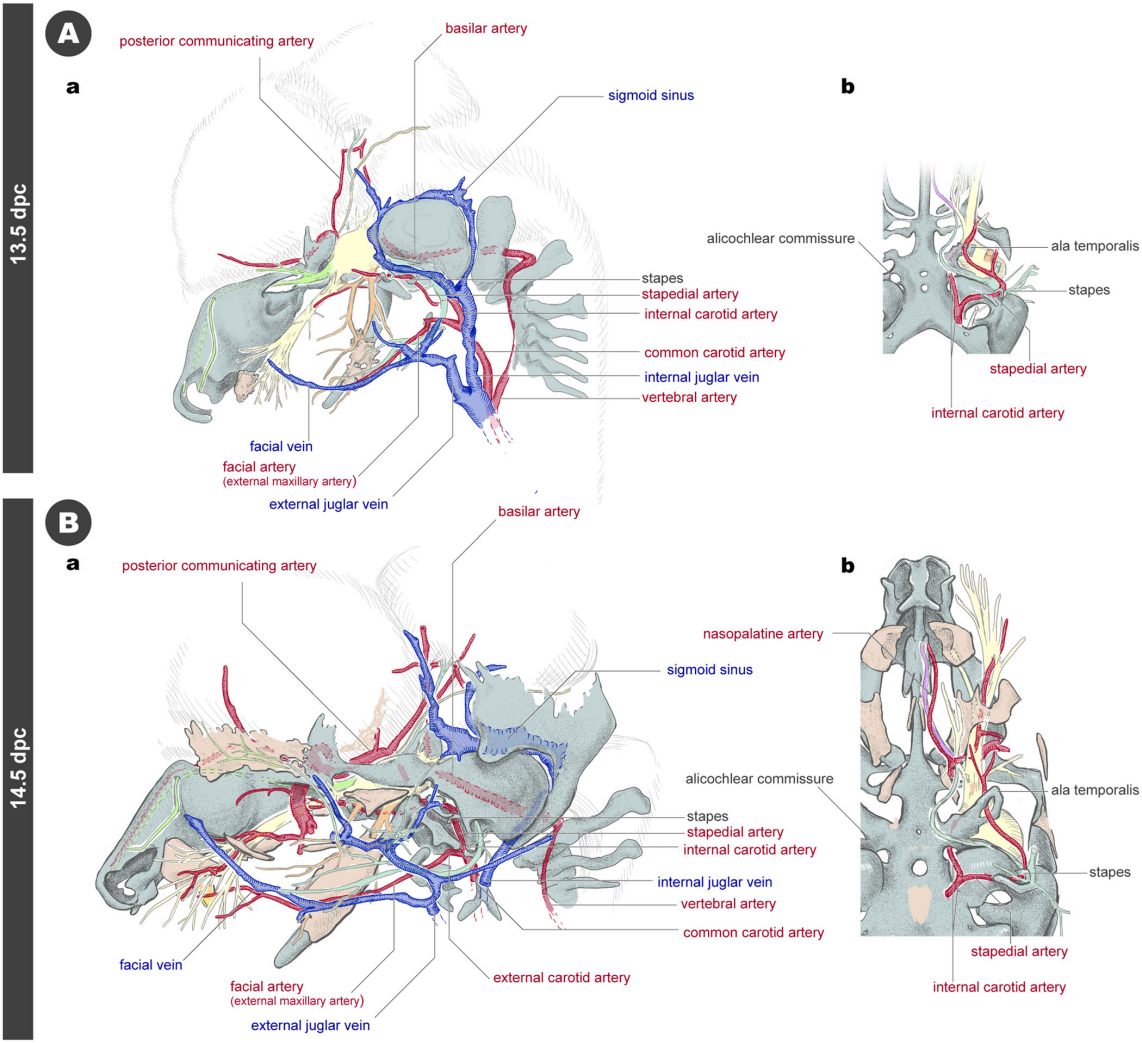
Arteries and veins at 13.5 and 14.5 dpc embryos. (A) The 13.5 dpc embryo. (a) Left lateral view. (b) Palatal view. (B) The 14.5 dpc embryo. (a) Left lateral view. (b) Palatal view.

At 13.5 dpc, the vascular system appears to undergo considerable remodeling (Figures 15A and S4). However, due to the difficulty of reconstructing the details from tissue sections in three dimensions, a detailed description of the arterial distribution in the upper jaw is omitted from this study. The chondrocranium forms around the internal carotid artery, leaving the surrounding space as the carotid foramen. The stapes forms in a ring encircling the stapedial artery (Figures 15Ab and S4). The facial venous system undergoes substantial changes, likely in correspondence with the development of facial musculature, which begins to cover the face. The facial vein extends from the face to the posterior part of the lower jaw (Figures 15A and S4).

By 14.5 dpc, further vascular remodeling occurs (Figures 15B and S5). The nasopalatine artery forms along the nasopalatine nerve in the palate (Figure 15Bb). Throughout this stage, the arteries and veins exhibit frequent reconnections, regressions, and branching as they develop in the maxillofacial region.

## 4 Discussion

In the present study, we have observed and described the morphogenesis of maxillofacial structures from the pharyngula to the fetal stage in mice. During the mid‐pharyngula stages (9.0‐9.5 dpc), the mouse head contains three distinct populations of cranial neural crest cells—trigeminal, hyoid, and circumpharyngeal crest cells—that migrate to specific premandibular and pharyngeal arch regions (Kuratani and Kirby 1991; Maden et al. 1992; Olsson and Hanken 1996; Horigome et al. 1999; Kuratani and Horigome 2000; Stundl et al. 2020. Reviewed by Kuratani 1996; Kuratani et al. 2018). These populations give rise to distinct branchiomeric nerves and associated ganglia (V, VII, IX, and X), with their spatial relationships being conserved throughout development (Gegenbaur 1871; Balfour 1877; van Wijhe 1882; Goodrich 1930; de Beer 1937; Kuratani 1996; Kuratani and Horigome 2000). Other features, such as the formation of the oropharyngeal membrane and hypophysis, also reflect highly conserved patterns across jawed vertebrates (see Patten 1927). This sharply contrasts with earlier developmental processes, like epiblast formation in mice, which differ markedly from other mammals and nonmammalian species (Sheng 2015; Christodoulou et al. 2019). In metazoan development, mid‐developmental stages are well known for exhibiting a high degree of similarity across species of the same phylum, both morphologically and genetically, a phase referred to as the phylotypic period, which is often illustrated as the thinnest part of an hourglass (Duboule 1994; Richardson 1995; Irie and Kuratani 2011; Hu et al. 2017). From a comparative morphological perspective, the mid‐pharyngula stages in mice can indeed be considered as fitting this phylotypic period, with the development of mammalian‐specific and mouse‐specific maxillofacial structures emerging as later modifications from this highly conserved pharyngula condition.

The following discussion focuses on maxillofacial development after the pharyngula stage, particularly emphasizing the cranial base and the much‐debated homology of the upper jaw bones. This discussion reviews and analyzes the unresolved issues in these regions and draws comparisons with humans and other vertebrates to establish a coherent morphological framework between mice and other jawed vertebrates.

### 4.1 Homology of the Hypophyseal Cartilage

The vertebrate cranium is derived from two primary developmental cell sources: cranial neural crest cells and mesodermal cells. Both cells form an organized cluster of undifferentiated cells, which collectively are called mesenchymal cells. These mesenchymal cells establish a distinct rostrocaudal boundary in the head that persists throughout development, as demonstrated by cell lineage tracing experiments in living jawed vertebrate embryos (Hörstadius and Sellman 1946; Couly et al. 1993; Olsson and Hanken 1996; McBratney‐Owen et al. 2008; McCarthy et al. 2016; Kuroda et al. 2021; 2023). In the cranial base, the rostral limit of the mesodermal mesenchyme is located at the hypophysis, adjacent to the tip of the notochord (McBratney‐Owen et al. 2008). Thus, the parachordal cartilage is entirely mesodermal in origin. Skeletal structures anterior to the hypophysis are derived from cranial neural crest cells, and the cranium from this region is usually termed the prechordal cranium (Gegenbaur 1871; Couly et al. 1993; Le Douarin and Kalcheim 1999). The most representative part of the prechordal cranium is the trabecular cartilage. Between the trabecular and parachordal cartilages, the hypophyseal cartilage (in therian mammals) or polar cartilage (in nonmammalian jawed vertebrates) often forms around the hypophysis, as also observed in mice (Figures 7 and S9).

The trabecular and parachordal cartilages are generally considered highly conserved across jawed vertebrates. In non‐mammals, the trabeculae are typically represented as a pair of rod‐like cartilages. In contrast, the left and right trabecular cartilages fuse along the midline to form the trabecular plate in mammals. This difference is thought to be associated with the platibasic morphology, where the cranial base is flattened (de Beer 1937). The homology between the hypophyseal and polar cartilages has long been debated.

In nonmammalian groups, such as chondrichthyans and birds, polar cartilages are a pair of chondrification centers that typically form lateral to the internal carotid arteries (de Beer and Woodger 1930; de Beer 1937). In contrast, hypophyseal cartilages in therian mammals are positioned medial to the internal carotid arteries, as observed in species like humans, cats, moles, rabbits, cattle, and armadillos (de Beer and Woodger 1930; MacPhee 1981). Based on the similarity of the cranial base, it is thought that the same applies to marsupials (Sánchez‐Villagra and Forasiepi 2017). This distinct position suggests that the hypophyseal cartilages in therian mammals occupy a different location compared to the polar cartilages in other jawed vertebrates, raising questions about which part of the mammalian chondrocranium is homologous to the polar cartilages (de Beer and Woodger 1930). de Beer (1937) proposed that the caudalmost parts of the trabecular and polar cartilages correspond to the alicochlear commissure, located lateral to the internal carotid artery in mammals (it first develops at 13.5 dpc, later than hypophyseal cartilage in mice, see Figure 15).

**Figure 16:**
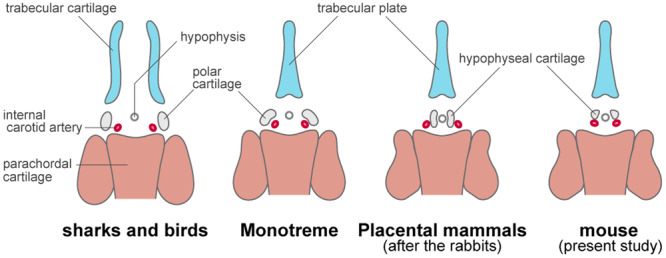
Homology of the cartilages comprising the cranial base. The schemes were redrawn from de Beer and Woodger (1930), except for the mouse, which is based on the present study. Color coding of the chondrocranium indicates the developmental origins, with blue indicating neural crest cells and orange indicating mesodermal origins. The origins of polar cartilage and hypophyseal cartilage (gray) are unknown.

Our observations in mice challenge the de Beer's scheme mentioned above. In mice, the trabecular plates and hypophyseal cartilages first become visible at 12.5 dpc (Figures 7, 14, and S3). At this stage, the internal carotid artery follows a complex three‐dimensional trajectory within the head, curving sharply near the hypophysis. In the same horizontal plane, the hypophyseal cartilages are located anterior to the internal carotid arteries, differing from what has been described in other therian mammals (Figure 16). The unique and divergent topographical relationship between the hypophyseal cartilages and internal carotid arteries in mammals is likely the result of gradual twisting during development. Previous research shows that, at 11.5 dpc, the internal carotid artery is positioned medial to the Sox9+ neural crest‐derived mesenchyme, while in later stages, it shifts into the mesodermal environment (Kuroda et al. 2023). This finding supports Gaupp's hypothesis, which proposed that the pathway of the internal carotid artery into the cranial cavity has been modified in therian mammals (Gaupp 1906). de Beer and Woodger (1930) primarily studied developmental stages after the hypophyseal cartilages had already fused with the trabecular and parachordal cartilages. Observations of earlier developmental stages in mammals might reveal that the hypophyseal cartilages are positioned more similarly to the polar cartilages in nonmammals.

Overall, there is no longer any reason to dismiss the homology between the hypophyseal cartilages in therian mammals and the polar cartilages in nonmammalian jawed vertebrates. Considering that these cartilages have traditionally been regarded as the caudalmost part of the trabecular cartilages, it is morphologically reasonable to hypothesize they originate from the neural crest cells. Yet, it remains uncertain whether these cartilages derive from neural crest cells or mesoderm, and further investigation is needed to clarify their true origin. Furthermore, recent cell lineage trace experiments using mice have demonstrated that chondrocranial elements such as the orbital cartilage and suprapterygoid articulation of the palatoquadrate, as well as the ala hypochiasmatica and supratrabecular cartilage, which were often doubted for their homology, are highly conserved in mammals and nonmammalian jawed vertebrates (Kuroda et al. 2023). Thus, it is reasonable to conclude that the most fundamental cranial pattern comprises highly conserved elements across jawed vertebrates, whether in mice or other nonmammalian species.

### 4.2 Chondrocranial Comparison of Mice and Men

**Figure 17:**
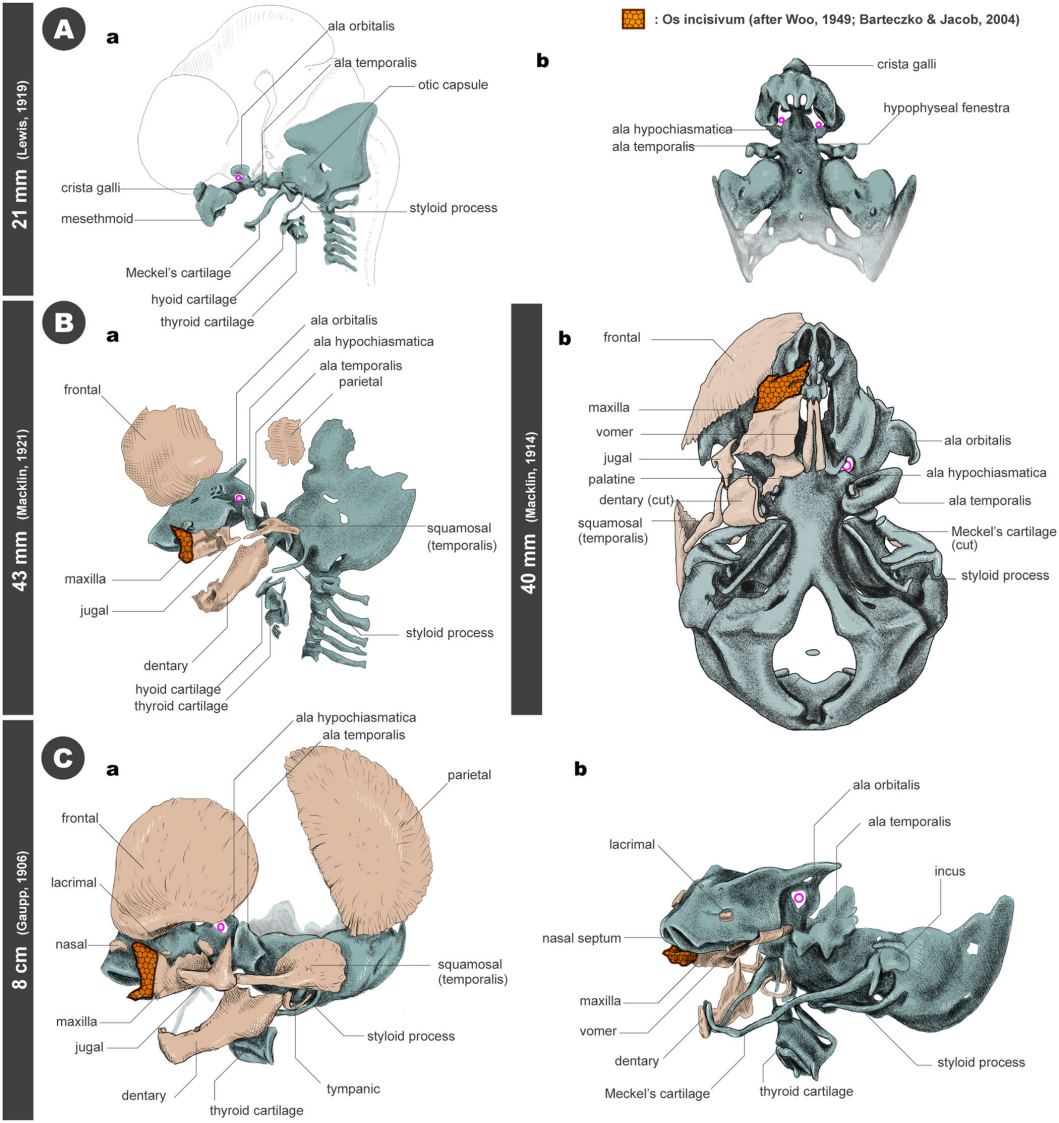
Development of the human skull. All schemes were redrawn from the classic literature. (A) The chondrocranium at a stage before the ossification begins. Redrawn from Lewis (1919). (a) Left lateral view. (b) palatal view. (B) The skulls slightly after the beginning of ossifications. (a) Left lateral view (redrawn from Macklin 1921). (b) palatal view (redrawn from Macklin 1914). de Beer and Woodger (1930). (C) The fetal skulls. Redrawn from Gaupp (1906). (a) Left lateral view. (b) Left lateral view and the left bony elements were removed. The parts surrounded in orange (mosaic texture) are the areas of the incisivum as inferred from the literature (Woo 1949; Barteczko and Jacob 2004).

The development of the human cranial skeleton has been well documented using several resources, such as the Carnegie Collections (see Figure 17; Macklin 1914; Lewis 1919; Macklin 1921). Based on our present data, the structure of the chondrocranium is highly conserved in mice. One notable difference is that the orbitoparietal commissure (taenia marginalis), which connects the nasal and auditory capsules in mice (Figures 9, 10, and S10), does not develop in humans. Consequently, no sphenoparietal fenestra is surrounded by the cartilage in humans, giving a seemingly different impression of the chondrocranium between the two species. The orbitoparietal commissure is generally present in most mammals, except for primates (e.g., *Nycticebus*, *Macacus*, and *Semnopithecus*) at the developmental stage when jaw ossification begins (de Beer 1937). The absence of this cartilaginous cranial wall in primates may correlate with the expansion of the cerebrum, especially the temporal lobe it encases.

Such morphological similarities allow us to infer the boundary between neural crest cells and mesodermal cells in the human skull. Several teams have already investigated the contribution of neural crest cells and mesoderm to the cranium in mice. In mice, as in other vertebrates, the position of the hypophysis serves as a landmark, with structures anterior to it originating from neural crest cells and those posterior to it derived from mesoderm (McBratney‐Owen et al. 2008; Kuroda et al. 2023). The only disagreement concerns the tectum transversum and alicochlear commissure.

**Figure 18:**
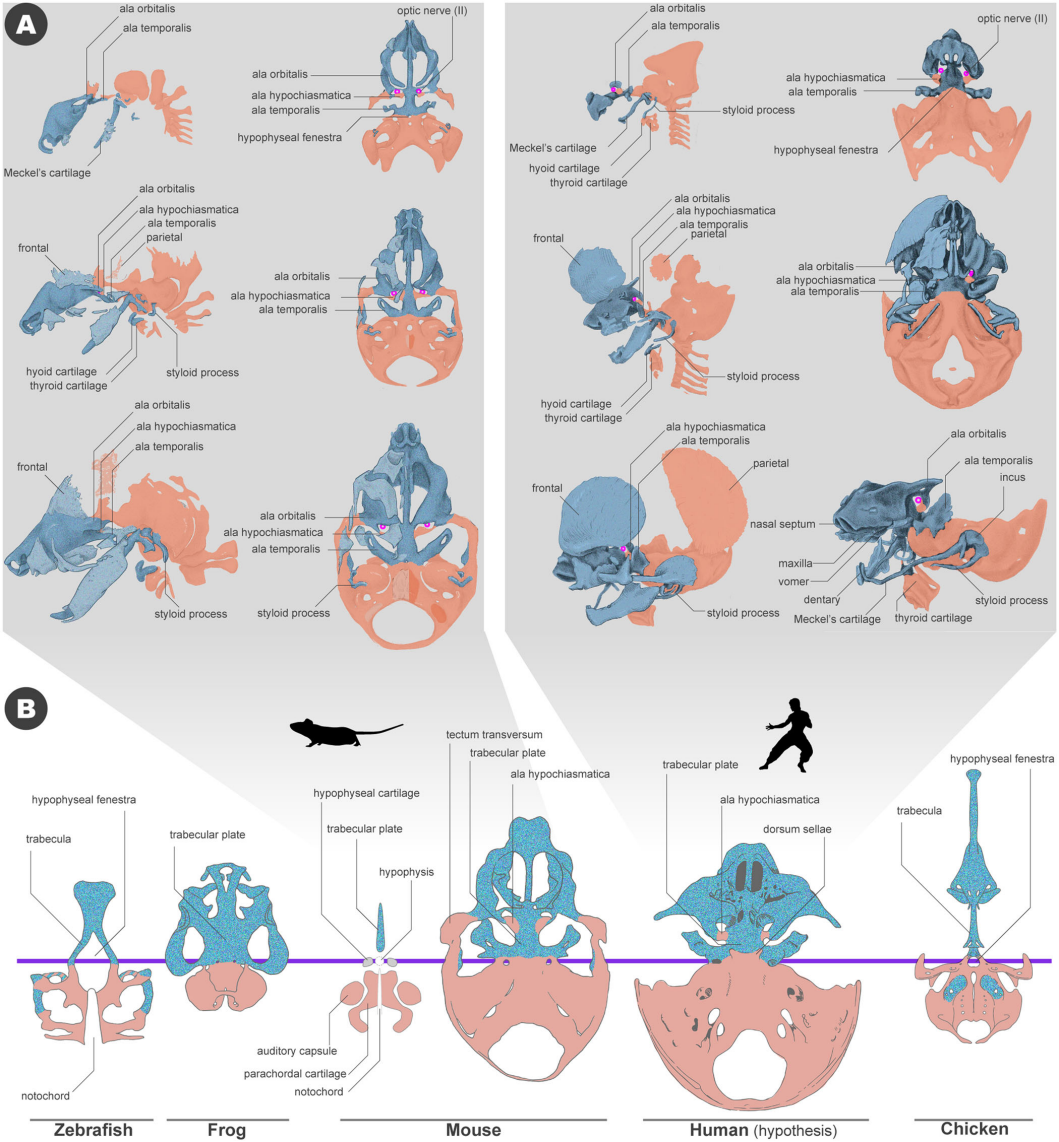
Developmental process of the cranial base and neural crest cell and mesodermal cell distributions. (A) The contributions of the cranial neural crest cells (blue) and mesoderm (orange) in mice and humans. The lineages of these cells in the mouse are based on Kuroda et al. (2023). The human cases were estimated based on comparing mouse data and the human cranial morphology shown in Figure 15. (B) Distribution of the cranial neural crest cells (blue with granular texture) and mesodermal cells (orange) derivatives in the chondrocranium of jawed vertebrates. Redrawn from Kuroda et al. (2023), except for Human (the outline is redrawn from Macklin (1921). All cranial bases are the dorsal views. The cellular contributions of chicken (Couly et al. 1993), frog (Olsson and Hanken 1996), and zebrafish (McCarthy et al. 2016) are based on the previous literature.

McBratney‐Owen et al. (2008) suggested that the tectum transversum and alicochlear commissure are derived from the neural crest cells, while Kuroda et al. (2023) and Pitirri et al. (2023) independently showed that they are from the mesodermal cells. Otherwise, there is considerable consensus regarding the neural crest or mesodermal origin of cranial skeletal structures (Figure 18). Although the ala hypochiasmatica is located rostral to the hypophysis, it is of mesodermal origin (McBratney‐Owen et al. 2008; Comai et al. 2020; Kuroda et al. 2023). This structure serves as the attachment site for all rectus components of the extraocular muscles in mammals and later becomes a part of the lesser wing of the presphenoid bone, along with the ala orbitalis, during development (McBratney‐Owen et al. 2008). It has been suggested that ala hypochiasmatica corresponds to the supratrabecula in the early chondrocranium of sauropsids (Kuratani 1987; Kuroda et al. 2023). Moreover, our whole‐mount skeletal staining shows a gap between the ala hypochiasmatica and the trabecular plate (Figure S10). Given that ala hypochiasmatica has a discrete embryonic origin from surrounding cartilaginous elements and its histological features, it is reasonable to consider this cartilage as an independent skeletal element. Based on the above discussion, the boundary between neural crest cells and mesoderm in the human cranial base should be summarized as shown in Figure 18.

The present suggestion should be helpful in the study of human cranial diseases. In the mice, the disruption of the boundary between neural crest cells and mesoderm in the cranium has been shown to cause a loss of the suture between the frontal and parietal bones, resulting in severe craniosynostosis (Ting et al. 2009; Tabler et al. 2016; Farmer et al. 2021). In human craniosynostosis, malformations also occur in the extraocular muscles, whereas the cause has not been clear (Greenberg and Pollard 1998; Tan et al. 2005). The proposed framework provides a hint to explain this; as seen in ala hypochiasmatica, the attachment site of the extraocular muscles is located at the boundary region between neural crest cells and mesodermal cells. Thus, disturbance of the boundary between the two cell groups has a high probability of causing considerable changes in the extraocular muscle attachment site formation.

The differences in the chondrocranium between mice and humans, or other typical mammals, are primarily quantitative. How these differences arise continues to be a topic of considerable debate today (Kaucka et al. 2017; Kyomen et al. 2023) and will likely remain a key focus for future research.

### 4.3 Incisivum as the Mammalian‐Unique Jaw Bone

**Figure 19:**
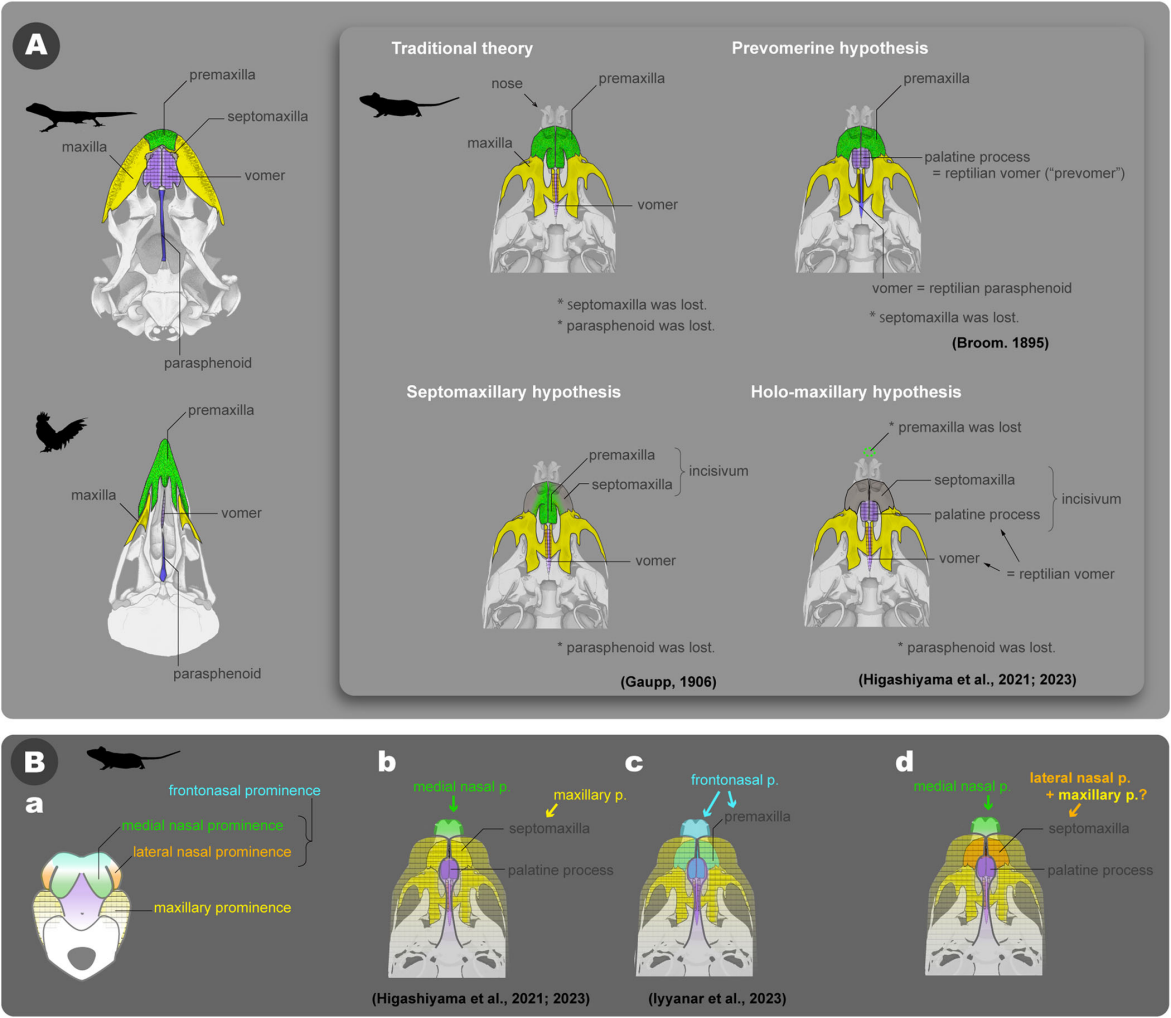
Hypotheses on the maxillofacial development of mammals. (A) Morphological homology of the upper jaw bones. The skulls of a lizard (*Paroedura picta*) and a chicken (*Gallus gallus*) are shown on the left. The septomaxilla is almost invisible from the palatal view, but its position is indicated for ease of comparison. The right panel shows diagrams from the palatal side of the murine skull, and illustrates four hypotheses: Traditional hypothesis (most common framework), Prevomerine hypothesis (Broom 1895), Septomaxillary hypothesis (Gaupp 1906), and Holo‐maxillary hypothesis (Higashiyama et al. 2021, 2023). (B) Developmental origin of the murine upper jaw. (a) The scheme of the palatal view of the mouse embryo. (b) Higashiyama's hypothesis (Higashiyama et al. 2021, 2023). (c) Iyyanar's hypothesis (Iyyanar et al. 2023). (d) A hybrid theory that explains the panels b and c without contradictions.

Despite the high conservation of the chondrocranial morphology, frequent challenges in comparing the facial features of mammalian and nonmammalian vertebrates lie in the homology of the rostral part of the upper jaw. Generally, the rostralmost tooth‐bearing bone is termed the premaxilla, while the main upper jaw bone, the maxilla, is located caudolaterally to the premaxilla. Although secondarily lost in chondrichthyans, this pattern of marginal jaw bones is generally considered highly conserved across almost all jawed vertebrates, including the maxillate placoderms (traditional hypothesis in Figure 19A; Zhu et al. 2013, 2016; Long 2016). However, the homology between the so‐called premaxillae in therian mammals and those in nonmammals has often been questioned.

Ernst Gaupp hypothesized that the incisivum of therian mammals is a composite structure comprising the premaxilla and the septomaxilla (Septomaxillary hypothesis in Figure 19Ac; Gaupp 1906). The septomaxilla is a small bone beside the nostrils in many nonmammalian tetrapods (except archosaurians) (Wible et al. 1990; Hillenius 2000). Gaupp observed the development of monotreme echidnas, in which the septomaxilla enlarged and covered the outer part of the nasal capsule, and he proposed that the facial process of the therian incisivum corresponds to this structure (Gaupp 1906). However, he did not find direct evidence for this in the incisivum of therian mammals.

Long before Gaupp's proposal, the complex nature of the incisivum as a composite of multiple bone elements had been suggested, partly due to observations of divided incisive bones in mammals with orofacial clefts, like cleft lip/palate (e.g., Albrecht 1879; Meyer 1884; Fawcett 1911; reviewed by Barteczko and Jacob 2004). Notably, Broom (1895) hypothesized that while the lateral body of the therian incisivum is the premaxilla, its palatine process has a different evolutionary origin—the vomer of nonmammalian amniotes (Prevomerine hypothesis in Figure 19Ab). According to Broom, the mammalian vomer is not homologous to the nonmammalian vomer. Instead, he proposed that the mammalian vomer originated from the nonmammalian parasphenoid, which is generally considered to be secondarily lost in mammals (Broom 1895). To avoid the confusion, he termed the nonmammalian vomer a “prevomer.” This prevomer hypothesis faced numerous objections over time (Parrington 1940; Atkins and Franz‐Odendaal 2016; Wible et al. 2018), and Gaupp (1906) dismissed it outright. However, de Beer (1937) appears to have found it worth further consideration, referring to the palatine process as the “prevomerine process”.

We recently suggested that the entire lateral body of the incisivum, excluding the palatine process, is homologous to the septomaxilla based on embryological and paleontological evidence (Higashiyama et al. 2021). In a follow‐up study, Higashiyama et al. (2023) suggested that the palatine process may correspond to the rostral half of the vomer in nonmammalian species (Figure 19Ad). Since the vomer has two distinct ossification centers in squamates and frogs (Smirnov and Vassilieva 2009; Sheverdyukova 2019; Nojiri et al. 2025), they hypothesized that the rostral ossification center became integrated into the incisivum. According to this hypothesis, the incisivum is a composite bone of nonmammalian septomaxilla and vomer, and the premaxillary region would have been completely lost in therian mammals (Holo‐maxillary hypothesis in Figure 19Ad).

The following discussion examines which of the four hypotheses mentioned above is most strongly supported by the data from this study. The incisivum begins ossifying by 13.5 dpc, located on the lateral side of the nasal capsule, just posterior to the narial fenestra of the chondrocranium. In the early stages of ossification, the incisivum laterally covers the anterior alveolar nerve, a branch of the maxillary nerve (V2), and the rostral end of the nasolacrimal duct (Figure 8B,D). The topographical position of this ossification center shows stark contrast to that of the nonmammalian premaxilla, which is located at the rostral tip of the chondrocranium, specifically in the cavum internasale (de Beer 1937; Bjerring 1978). This area receives the medial nasal branch of the V1 nerve, while the alveolar nerve, along with the nasolacrimal duct, is associated with the septomaxilla (Higashiyama et al. 2021). In mice, no bony element forms at the position corresponding to the premaxillary ossification centers of the nonmammalian tetrapods, and the rostralmost part of the neurocranium differentiates into nasal cartilage (Figures 8, 9, 10, 11, 12). This topographical difference should not be due to developmental distortion, as it would also affect the surrounding soft tissues. In nonmammalian species (except crocodilians), the premaxilla always corresponds to the distribution area of the medial nasal branch of V1 (Figure S12; Higashiyama and Kuratani 2014, 2021, 2023). If the ossification center of the premaxilla had shifted to the location of the incisivum due to secondary distortion, the mesenchyme, including the medial nasal branch, would also have shifted accordingly. However, the incisivum in mice is located within the territory of the maxillary nerve, V2, while the medial nasal branch continues to pass through both sides of the septal cartilage and terminates at the rostral end of the nose (Figure S12). Thus, based on their topographical relationships, the lateral body of the incisivum should not be considered homologous to the premaxilla of nonmammalian tetrapods.

The palatine process is first observed in mice at 15.5 dpc, connected to the main body of the incisivum (Figure 11). But, since the palatine process develops independently in many mammals, such as bats, goats, and armadillos (Higashiyama et al. 2023 and references therein), it is reasonable to consider it a separate bone element. Notably, with regard to peripheral nerves, the palatine process ossifies within the distribution area of the nasopalatine nerve. Anatomically, the nasopalatine nerve is typically considered a branch of V2, but it has a different origin from a developmental perspective. Namely, the nasopalatine nerve branches from an anastomosis between the palatine branch of the facial nerve (VII) and V2, although most V2 nerves are distributed to the maxillary prominence from the pharyngula stage. The nasopalatine nerve passes through the base of the trabecular plate at an early developmental stage and eventually innervates the primary palate (Figures 6, 7, S7, and S8; Higashiyama et al. 2014). This suggests that the nasopalatine nerve's region and the distribution areas of other V2 branches originate from different developmental primordia. The fact that the palatine process, like the vomer, develops within the nasopalatine nerve's distribution area suggests that these bone elements should originate from the same embryonic domain, which should be different from the rest of the incisivum with V2. Furthermore, the position of the palatine process during development—ventral to the paraseptal cartilage and thus ventral to the vomeronasal organ—mirrors the location of the anterior part of the vomer in squamates, further suggesting their homology.

Our present observation supports the Holo‐maxillary hypothesis by Higashiyama et al. (2021, 2023): during mammalian evolution, the ancestral premaxilla gradually disappeared, leaving the rostralmost part of the neurocranium as exposed nasal cartilage. Concurrently, the incisivum emerged as a new jaw bone formed from the septomaxilla and parts of the ancestral vomer. This challenges the widely accepted notion that the incisive bone in therian mammals is homologous to the premaxilla in nonmammalian species.

### 4.4 Developmental Origins of Incisivum

As demonstrated in the cell lineage tracing experiments using chickens (Lee et al. 2004), the formation of the upper jaw in nonmammalian tetrapods involves contributions from the medial nasal prominence to the rostromedial part, which gives rise to the premaxilla, while the maxillary prominences form the caudolateral portions of the upper jaw (Lee et al. 2004; Higashiyama and Kuratani 2014). In contrast, according to the holo‐maxillary hypothesis discussed in the previous section, a drastic reorganization of facial prominences is suggested to have occurred during mammalian evolution. With the loss of the premaxilla and a shift in this region from the upper jaw‐tip to form the nose, nearly the entire upper jaw in mammals is expected to derive from the maxillary prominences (Higashiyama et al. 2021, 2023).

Experiments in mice actually have shown that the mammalian upper jaw region is almost entirely composed of the maxillary prominence, except for the primary palate. *Dlx1* is specifically expressed in the mesenchyme of the pharyngeal arches during the mid‐pharyngula stage (Depew et al. 2002). Thus, *Dlx1*‐CreER^T2^ mice are expected to label the mandibular arch and its derivation, such as the maxillary prominence mesenchymal cells, and distinguish it from the premandibular mesenchymal cells. As the results of the cell lineage tracing experiments with them, *Dlx1* ^+^ cells were found throughout the upper jaw—except in the primary palate—across developmental stages, suggesting that the maxillary prominence extends to the rostralmost part of the murine upper jaw (Higashiyama et al. 2021, 2023; Iyyanar et al. 2023). This evidence implies that the mammalian face evolved through considerable reorganization of the otherwise conserved topographical relationships among facial prominences.

The developmental origin of the Os incisivum, however, requires careful consideration. While *Dlx1*‐CreER^T2^ mice have shown labeling in this bone, indicating a contribution from the maxillary prominence (Higashiyama et al. 2021, 2023), lineage tracing experiments using *Alx3*‐CreER^T2^ mice —which label mesenchyme from the premandibular domain—have also detected marked cells within the incisivum exclusively (Iyyanar et al. 2023; Figure 19Bc). Consequently, while consensus suggests that most upper jaw structures derive from the maxillary prominence, contrasting results regarding the origin of the incisivum raise the possibility of derivation from either the maxillary prominence or the premandibular domain.

Here, we provide insight into the above inconsistency based on our morphological observations. As with the robust correspondence of branchiomeric nerves and pharyngeal arches, the distribution of each major branch of the trigeminal nerve generally aligns with the facial prominences. The V1 nerve is associated with the premandibular domain, while the V2 and V3 nerves are associated with the maxillary and mandibular prominences, respectively. The medial and lateral nasal branches of the V1 nerve correspond to the medial and lateral nasal prominences. In actuality, the premaxilla derived from the medial nasal prominence corresponds to the distribution of the medial nasal branch of the V1 nerve in nonmammalian tetrapods (Figure S11; Higashiyama and Kuratani 2014). In mice, V2 and V3 nerves correspond to the maxillary and mandibular prominences, respectively (Figure 5Ba,b). The facial prominences remain distinct until 12.5 dpc, and the V2 nerve is extending throughout the maxillary prominence (Figures 4 and 5). This nerve distribution corresponds with the labeled cells observed in *Dlx1*‐CreER^T2^ mice (Higashiyama et al. 2021, 2023). By 13.5 dpc, the ossification of the incisivum begins within the V2 nerve distribution area (Figure 8). It is never associated with the V1 nerve, especially not with the medial nasal branch. Thus, given the correspondence between the nerves and the facial prominence mesenchyme and their continuous development, it is plausible that the lateral body of the incisivum originates within the environment of the maxillary prominence.

Consideration of cleft lip/palate embryos underscores the above discussion. In typical cases of cleft lip/palate, a physical gap forms between the medial nasal prominence and the maxillary prominence (Inouye 1912; Kondo 1994; Mossey et al. 2009; Dixon et al. 2011; Leslie and Marazita 2013). In diapsids, such as lizards, chickens, and snakes, the medial nasal branch of V1 and the premaxilla develop on the medial side of this gap, specifically within the medial nasal prominence (Bellairs and Boyd 1957; Bellairs 1965; Higashiyama et al. 2021). In contrast, in mice, the lateral body of the incisivum ossifies on the lateral side of the gap, indicating that it should not be derived from the medial nasal prominence, unlike the nonmammalian premaxilla (Higashiyama et al. 2021, 2023). Note that the palatine process also remains on the medial side of the cleft (although it disappears in some mutants; Heyne et al. 2015). But, it is located within the distribution area of the nasopalatine nerve, unlike the nonmammalian premaxilla (Figures 7, 8, and 10A; Higashiyama et al. 2021, 2023). This suggests that the ossification center of the palatine process is explicitly associated with the primary palatal region, corresponding to the palatal side of the medial nasal prominence.

Based on the above, it is most plausible to regard the lateral body of the incisivum as arising morphologically within the maxillary prominence. Given its topographical relationship with nerves and perspectives from cleft lip/palate studies, the structure is unlikely to derive from the medial nasal prominence like the nonmammalian premaxilla. Still, the possibility remains that cells from the lateral nasal prominence contribute to the incisivum. In tetrapods with cleft lip/palate, usually, there are no gaps between the lateral nasal and maxillary prominences, and the boundaries between them cannot be precisely identified. Furthermore, the ossification center of the lateral body is also adjacent to the nasolacrimal duct, which originates from the nasolacrimal groove, the boundary between the maxillary and lateral nasal prominences (Figures 6 and 8B,D), as with the septomaxillae of the nonmammalian tetrapods (Hillenius 2000; Higashiyama et al. 2021). Thus, even within the maxillary prominence, the incisivum should form along the boundary between these facial prominences, possibly coexisting cells from both the maxillary and lateral nasal prominence.

It is currently impossible to determine whether the maxillary or lateral nasal prominence contributes more significantly to the lateral body of the incisivum. In *Dlx1*‐CreER^T2^ mice, labeled cells within the incisivum were sparse compared to the more proximal part of the upper jaw (Higashiyama et al. 2021, 2023; Iyyanar et al. 2023). This pattern should reflect the uneven expression pattern of the *Dlx1* gene within the maxillary prominence at 10.5 dpc, with fewer expressing cells in distal regions (Jeong et al. 2008; Higashiyama et al. 2021, 2023) rather than suggesting a minimal contribution from the maxillary prominence. Moreover, the morphological system of facial prominence and cellular lineage do not completely overlap, especially in the early stages of prominence formation. Lineage tracing experiments in chickens have suggested that during the development from the mid‐pharyngula stage with an undifferentiated mandibular arch, cells from the premandibular domain contribute to the distal end of the budding maxillary prominence as development proceeds (Lee et al. 2004). Similarly, during the pharyngula stage in mice, perhaps the maxillary prominence contains cells at its distal end from a boundary region that cannot be definitively classified as either the mandibular arch or the premandibular domain.

Facial prominences, such as the maxillary prominence, represent highly conserved developmental modules from a comparative morphological perspective. However, the mechanisms by which these developmental primordia maintain their identity while incorporating temporally asymmetrical cell lineages during development remain incompletely elucidated. Recent advancements in spatiotemporal transcriptomics have enabled remarkably detailed and comprehensive analyses of embryonic development (Chen et al. 2022; Qiu et al. 2024). It is anticipated that these technological developments will give rise to novel definitions of developmental modularity, particularly for primordia‐like facial prominences that encompass multiple anatomical elements.

In summary, the most reasonable hypothesis is that the lateral body of the murine incisivum and the surrounding upper jaw soft tissues are derived from maxillary prominence at the organ level. At the tissue level, some neural crest cells from the lateral nasal prominence may intermingle within the maxillary prominence and contribute to the incisivum formation. The results of the lineage tracing experiments (Higashiyama et al. 2021, 2023; Iyyanar et al. 2023) are not mutually exclusive but rather support the complex nature of the incisivum or the septomaxilla (Figure 19Bd). Contributions from the medial nasal prominence appear unlikely, indicating that the lateral body of the incisivum should not be directly equated with the premaxilla. Although largely overlooked in recent research, the number of ossification centers in the mammalian incisivum and their facial prominence origins has long been a complex and debated issue, with numerous hypotheses previously proposed (Albrecht 1879; Warynski 1888; Meyer 1884; Inouye 1912; Jarmer 1922). Also, a recent study suggested that the facial prominence development mechanisms characterized in chickens may actually be highly specialized (Marchini et al. 2025). Further detailed research, especially cell lineage tracing, will be essential for understanding the evolution and development of the rostralmost upper jaw.

### 4.5 The Maxillofacial Scheme of Mice and Men

“The best‐laid schemes of mice and men/Go oft awry”— penned by Robert Burns in *To a Mouse* (1785) to express sympathy for a field mouse—aptly applies to the anatomical scheme of the rostral upper jaw. The incisivum has frequently been a subject of debate when comparing humans with other mammals. In adult humans, the hard bone that constitutes the upper jaw seems to be comprised solely of a pair of maxillae. As famously known, the incisivum (sometimes referred to as the premaxilla, intermaxilla, or Zwischenkieferknochen) arises during development even in humans and fuses with the maxilla (Coiter 1573; Goethe 1784/1831; Leuckart 1840). Reviewed by Barteczko and Jacob 2004). But there is no clear answer to the debate about where the boundary between the human incisivum and maxilla is or how many ossification centers there are in the incisivum itself (Albrecht 1879; Meyer 1884; Ranke 1901; Fawcett 1911; Inouye 1912; Jarmer 1922; reviewed by Barteczko and Jacob 2004).

Comparisons between humans and other mammals present challenges in cases of congenital abnormalities such as cleft lip/palate. In typical bilateral cleft lip/palate in humans, it is generally recognized that the entire incisivum forms medially between the clefts, while the maxilla develops laterally (Inouye 1912; Bøhn 1963; Orr et al. 2016). Variations in the position of the incisivum relative to the cleft in the face can also be seen in the diverse facial morphology of bats (Orr et al. 2016; Usui and Tokita 2018; Meguro et al. 2024). As discussed in the above sections, in mice, the incisivum consists of the palatine process and the lateral body, with each distributed medially and laterally relative to the clefts, respectively. This feature of the incisivum is also true in other nonhuman mammals (Albrecht 1879; Jin et al. 2012), including the macaque monkeys (Wei et al. 2000). Furthermore, in the human cleft lip/palate, the medial bony element is exclusively innervated by the nasopalatine nerve, and the nasopalatine artery surrounds it (Bøhn 1961, 1963), just like as the palatine process of mice. Thus, in human cases of cleft lip/palate, it should be regarded that the medial bony element corresponds to an enlarged palatine process, while the lateral body of the incisivum fuses with the maxilla on the lateral side of the cleft.

If the interpretation discussed above is valid, then in cleft lip/palate humans, the palatine process, which normally does not bear teeth, becomes excessively enlarged and contains incisors (Inouye 1912; Bøhn 1963). It is essential to note that tooth formation originates from the epithelial dental placode, with attachment to the jawbone, forming the dental alveolus at a later stage of development. The primary dental placode initially forms in a horseshoe shape across the medial nasal prominence and maxillary prominence (at 10.5 dpc in mice and 13.5 dpc in rats; Kriangkrai et al. 2006). As development progresses, the facial prominences fuse to form the upper jaw. In rodents, this fusion reduces the number of dental placodes, but in cleft lip/palate cases, the placodes remain on each prominence (Kriangkrai et al. 2006). Thus, the position and number of teeth vary depending on the condition of the facial prominences. Since the V2 nerve innervates the upper jaw, normal development of the human upper jaw should derive primarily from the maxillary prominence, likely including the philtrum. When the prominences fail to fuse, signals inducing the primary dental placode persist in the medial nasal prominence, forming incisors on the enlarged palatine process. Furthermore, it is also known that nerves serve as providers of multipotent neural crest‐like cells, including tooth progenitor cells (Kaucká and Adameyko 2014; Adameyko and Fried 2016), suggesting that alterations in nerve distribution due to abnormalities in facial prominence formation could subsequently affect bone development and ectopic teeth formation. Even if a pair of incisor teeth are present medially to the clefts in the cleft lip/palate humans, this does not necessarily imply a morphological boundary between the first and second incisors in a normal adult. The malformed morphology should be understood as a result of alterations in the embryonic developmental system.

**Figure 20:**
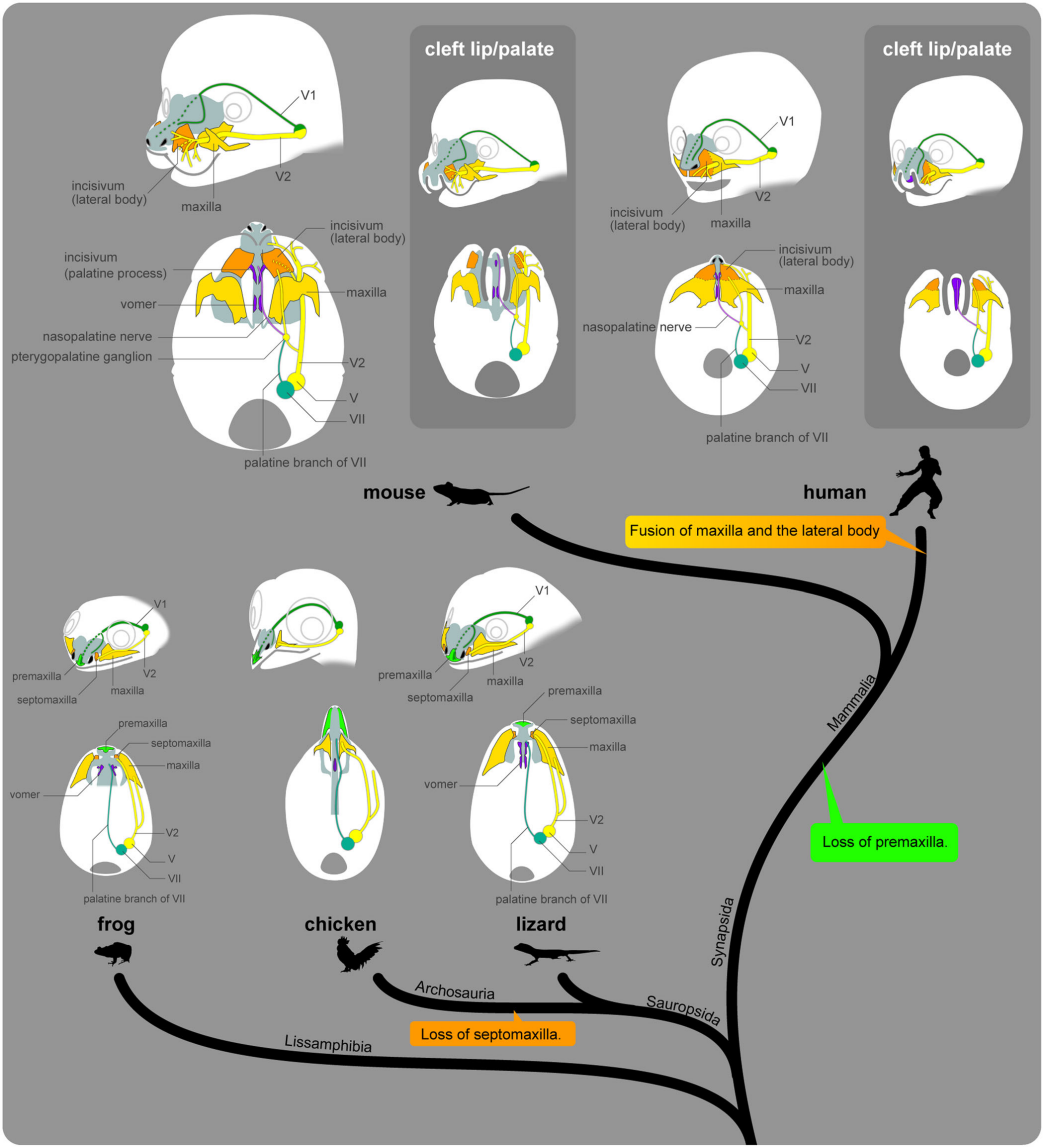
Schemes of the maxillofacial morphology among tetrapods. The schemes of the cleft lip/palate in mouse, lizard, chicken, and frog are based on the holo‐maxillary hypothesis from our previous study (Higashiyama et al. 2021, 2023). The cleft lip/palate in human was drawn after Inouye (1912) and Bøhn (1963). The nasopalatine nerve innervates primary palate in mammals, and the facial branch of VII is distributed in the homologous region in nonmammalian tetrapods. This is because V and VII do not anastomose at the pharyngula stage in the nonmammalian embryos (see Higashiyama and Kuratani 2014).

To summarize the above discussions, the evolutionary morphological scheme of the maxillofacial region is illustrated in Figure 20. Throughout mammalian evolution, the arrangement of ancestral facial prominences underwent considerable reorganization to form the holo‐maxillary upper jaw. This morphological pattern is shared across mammals, including in the maxillofacial anatomy of humans.

In conclusion, although mice possess highly derived features in their heads, such as enlarged incisors, their basic morphogenesis of the cranial skeleton, nerves, and vascular systems can still be considered typical of therian mammals. The present study provides a unified framework of the cranial base and the maxillofacial region for comparing humans with nonmammalian model organisms, such as chickens, using mice as an intermediary. Research on the morphology of mice remains indispensable for deepening our morphological understanding of mammalian evolution and human disease.

## Author Contributions

**Hiroki Higashiyama:** conceptualization, methodology, software, data curation, investigation, validation, supervision, funding acquisition, visualization, resources, project administration, writing–review and editing, writing–original draft, formal analysis. **Shunya Kuroda:** validation, visualization, writing–review and editing. **Akiyasu Iwase:** data curation, resources, writing–review and editing. **Naoki Irie:** writing–review and editing, validation. **Hiroki Kurihara:** funding acquisition, writing–review and editing, resources, validation.

### Peer Review

The peer review history for this article is available at https://www.webofscience.com/api/gateway/wos/peer-review/10.1002/jmor.70032.

## Supporting information

Supporting information.

## Data Availability

The data that support the findings of this study are openly available in SSBD:repository (https://ssbd.riken.jp/repository/) https://doi.org/10.24631/ssbd.repos.2025.01.413.
