# Supplementary material for: On the Maxillofacial Development of Mice, Mus musculus
Source: J Morphol. 2025 Feb 28;286(3):e70032. doi: 10.1002/jmor.70032 (PMC11871421; doi:10.1002/jmor.70032)
Supplement: Supplementary file 1 — Supporting information. [file JMOR-286-e70032-s001.pdf]

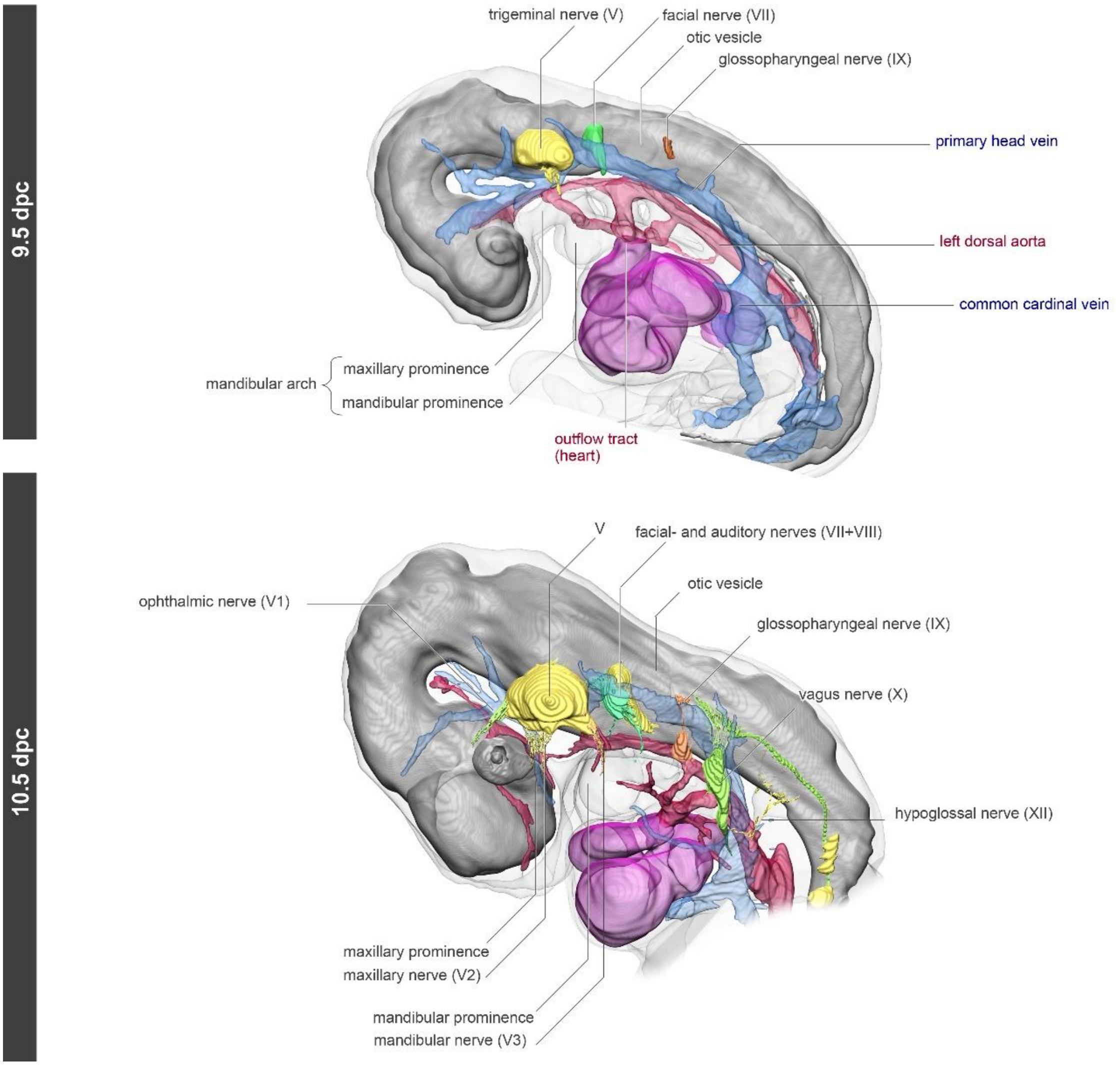

Figure S1. Three-dimensional models of the mouse embryos at 9.5 dpc and 10.5 dpc.

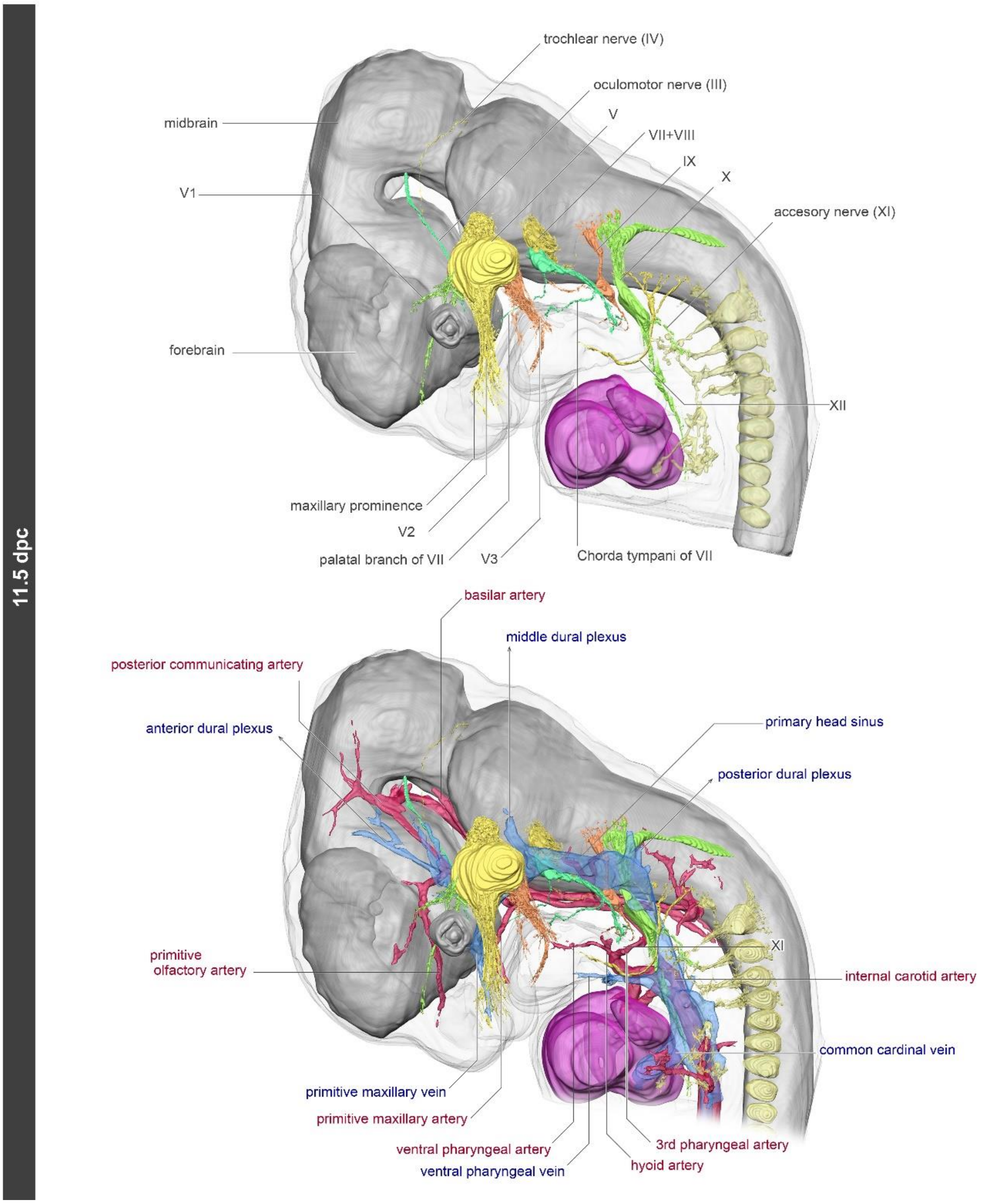

Figure S2. Three-dimensional models of the mouse embryos at 11.5 dpc.

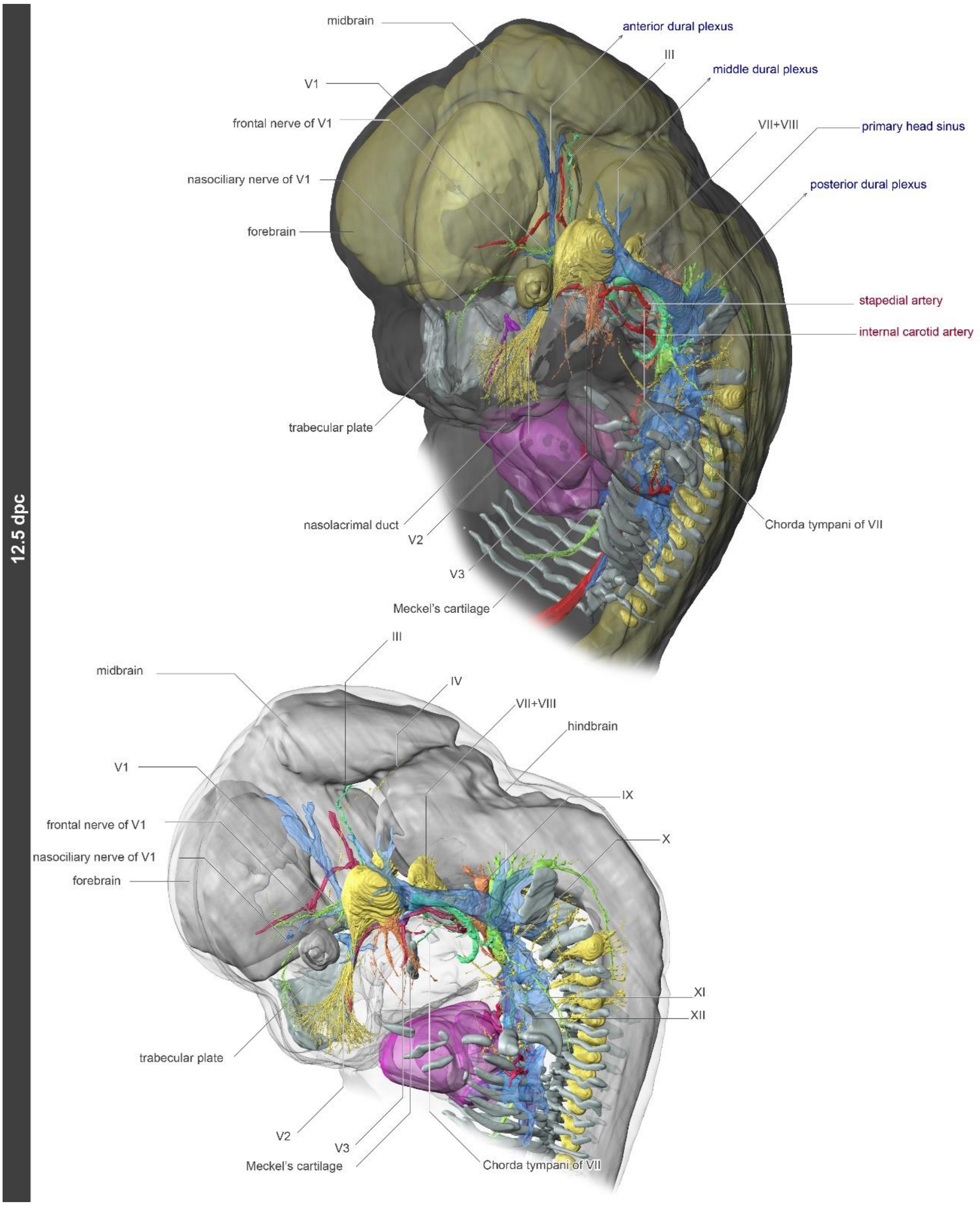

Figure S3. Three-dimensional models of the mouse embryos at 12.5 dpc.

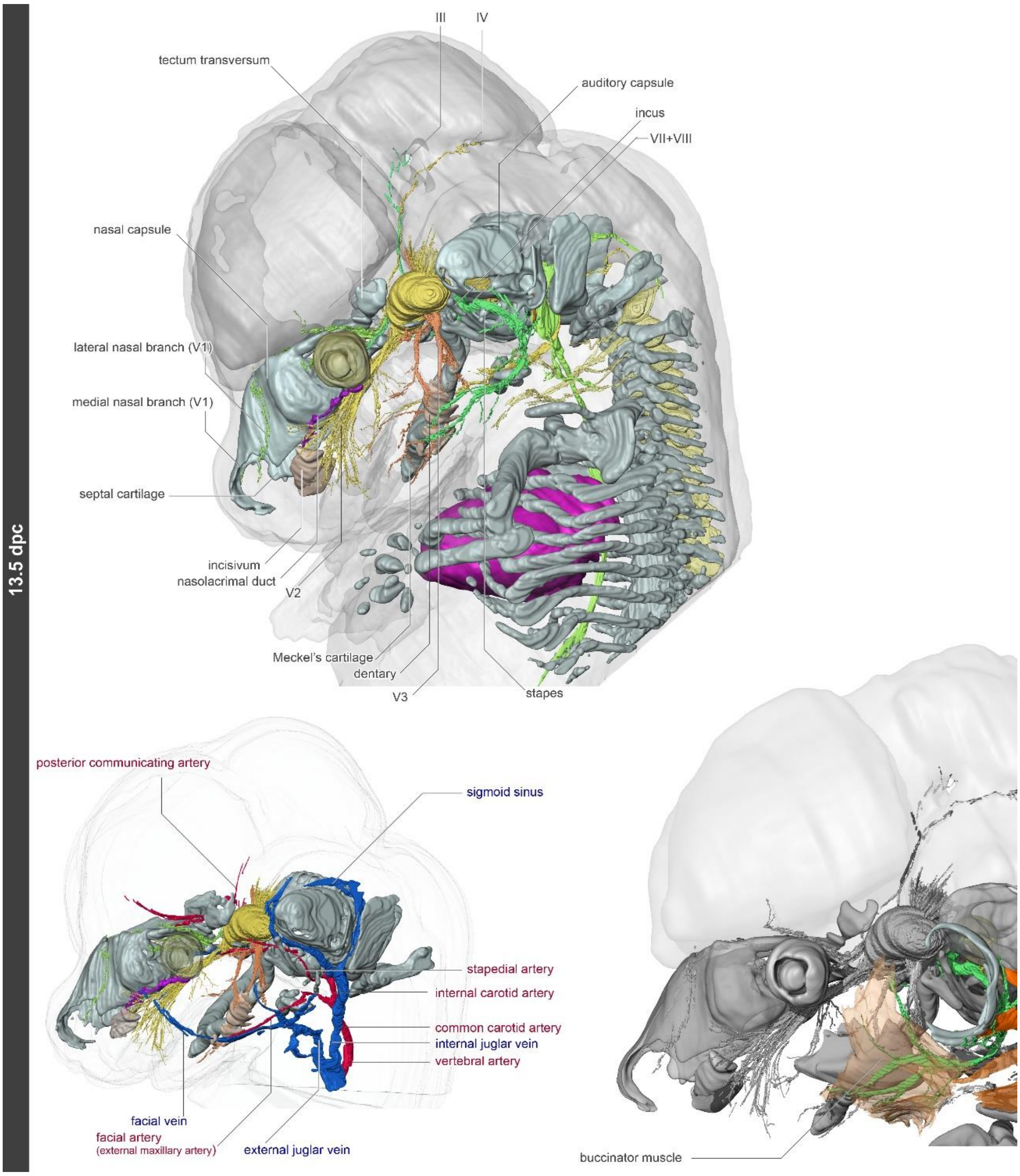

Figure S4. Three-dimensional models of the mouse embryos at 13.5 dpc.

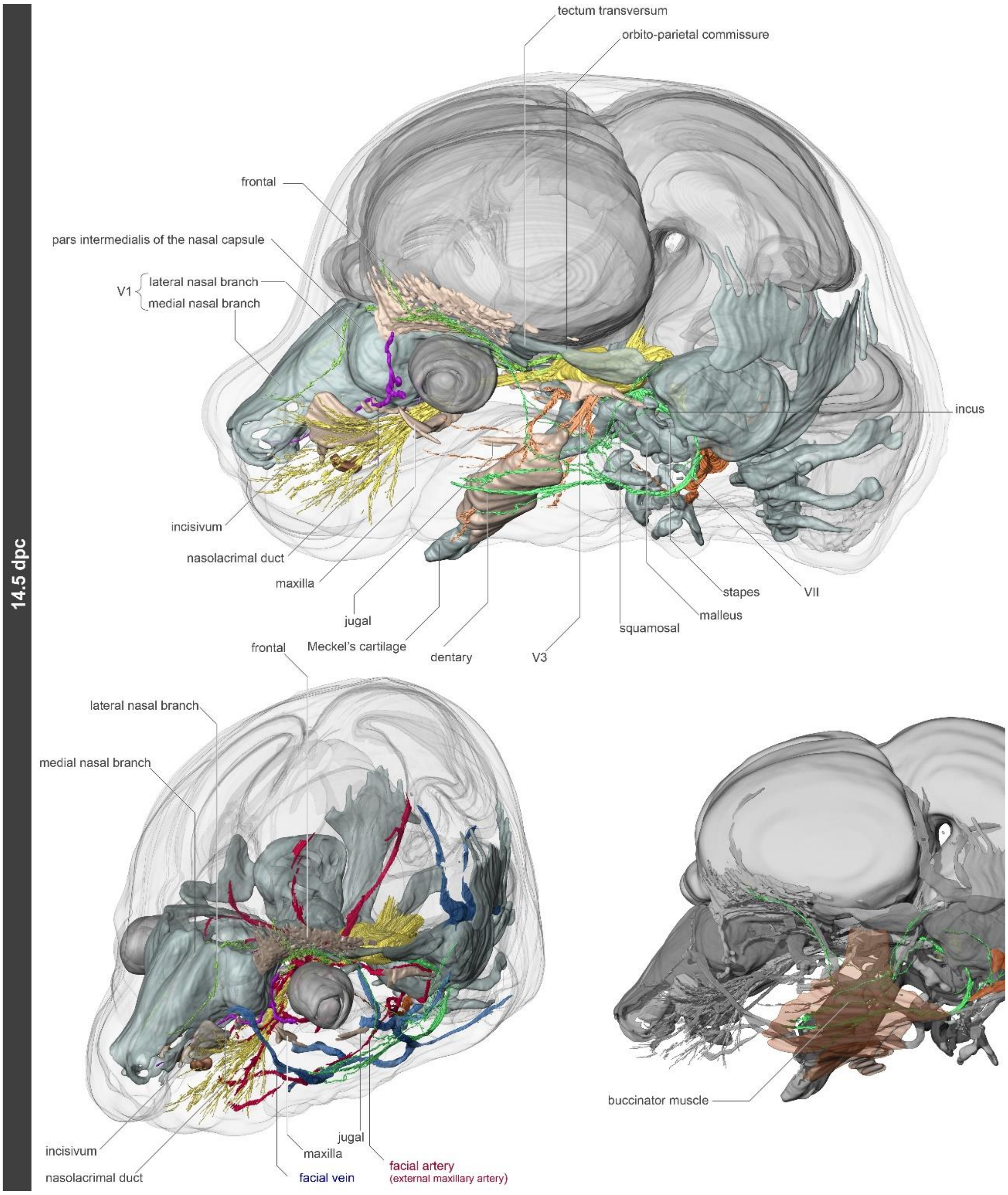

Figure S5. Three-dimensional models of the mouse embryos at 14.5 dpc.

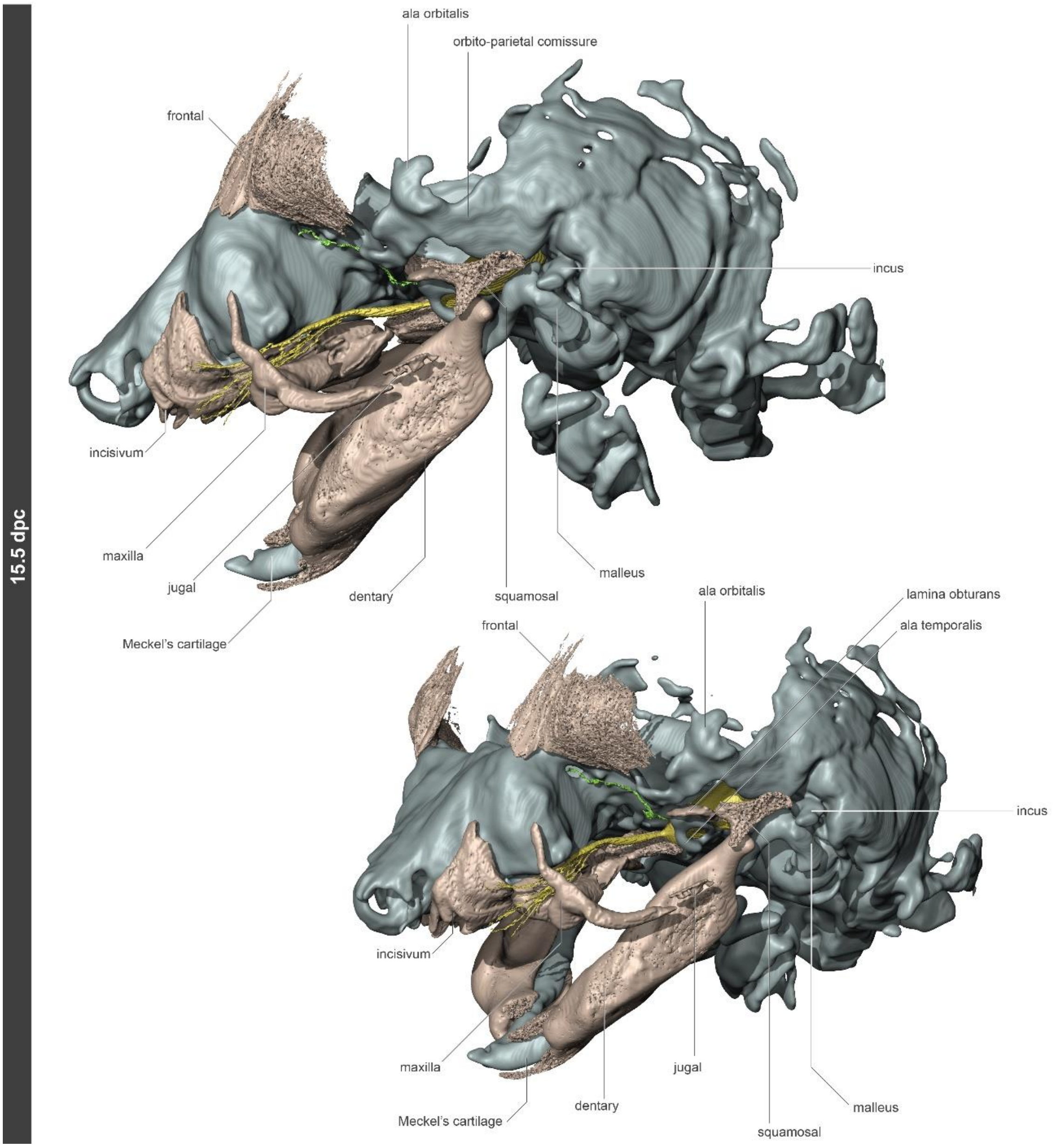

Figure S6. Three-dimensional models of the mouse embryos at 15.5 dpc.

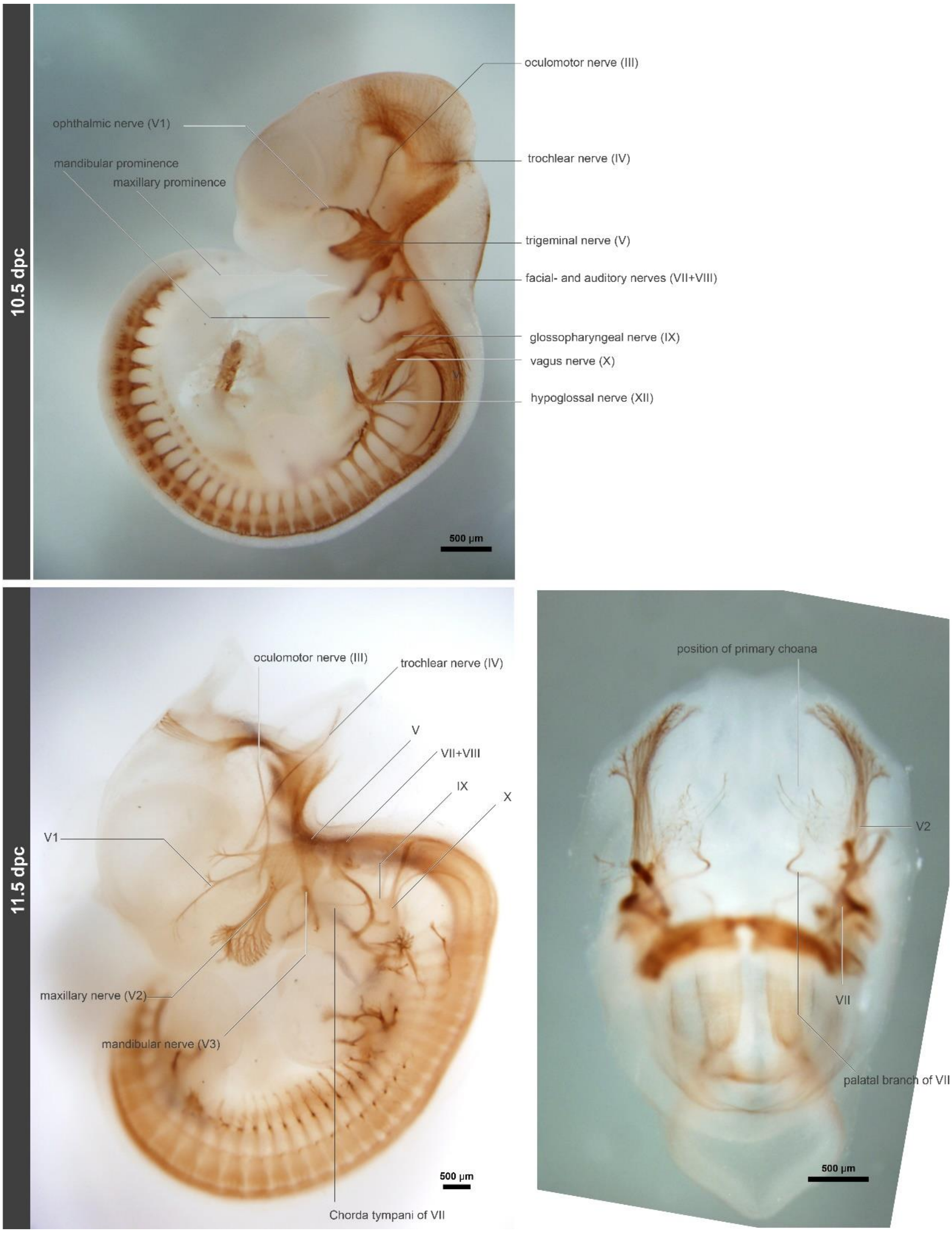

Figure S7. Immunohistochemistry of the neurofilament in 10.5 and 11.5 dpc mice.
We used monoclonal antibody 2H3 (Developmental Studies Hybridoma Bank) for visualizing the neurofilaments. For detail, also see Higashiyama and Kuratani (2014).

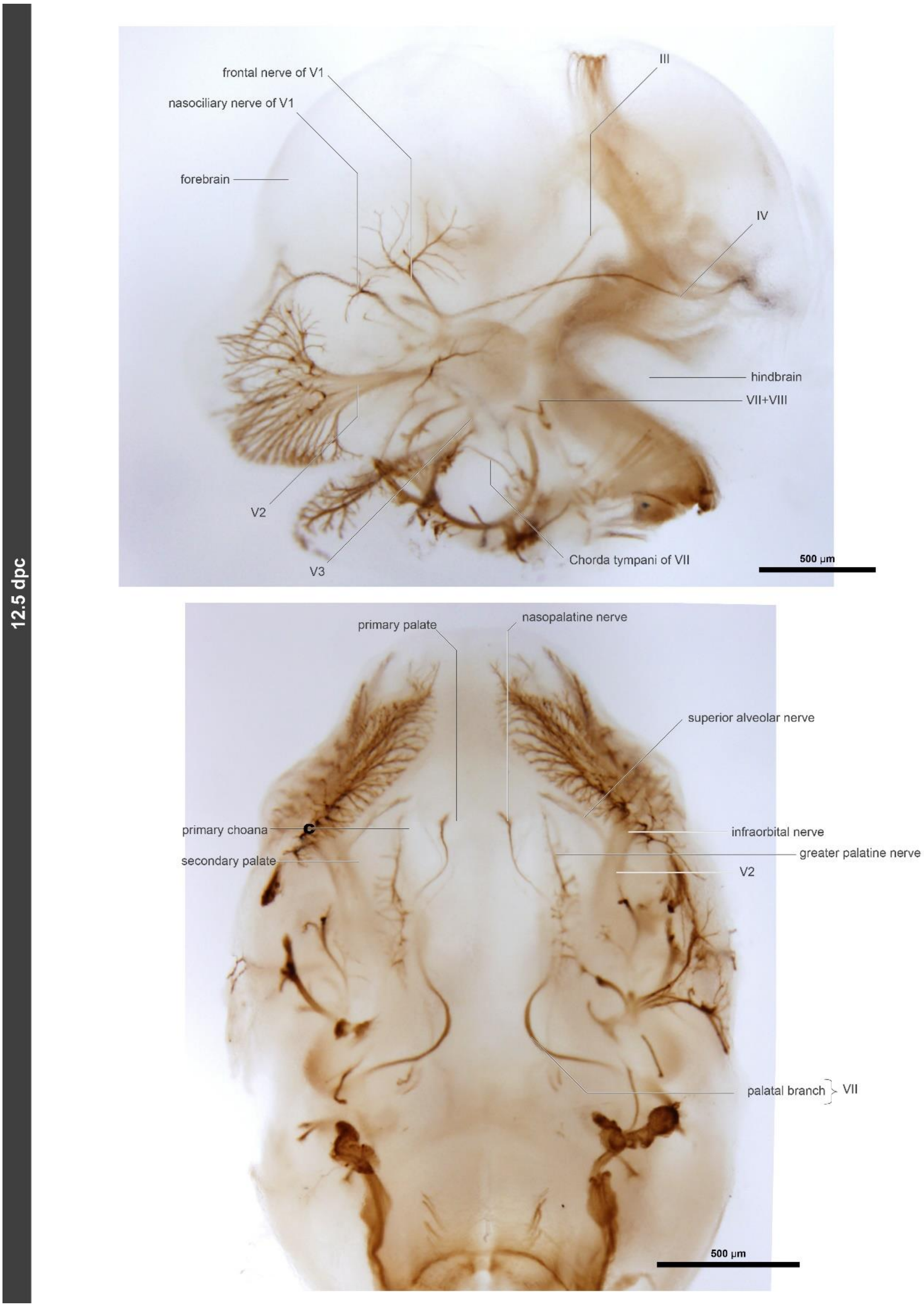

Figure S8. Immunohistochemistry of the neurofilament in 12.5 dpc mouse embryo.
We used monoclonal antibody 2H3 (Developmental Studies Hybridoma Bank) for visualizing the neurofilaments. For detail, also see Higashiyama and Kuratani (2014).

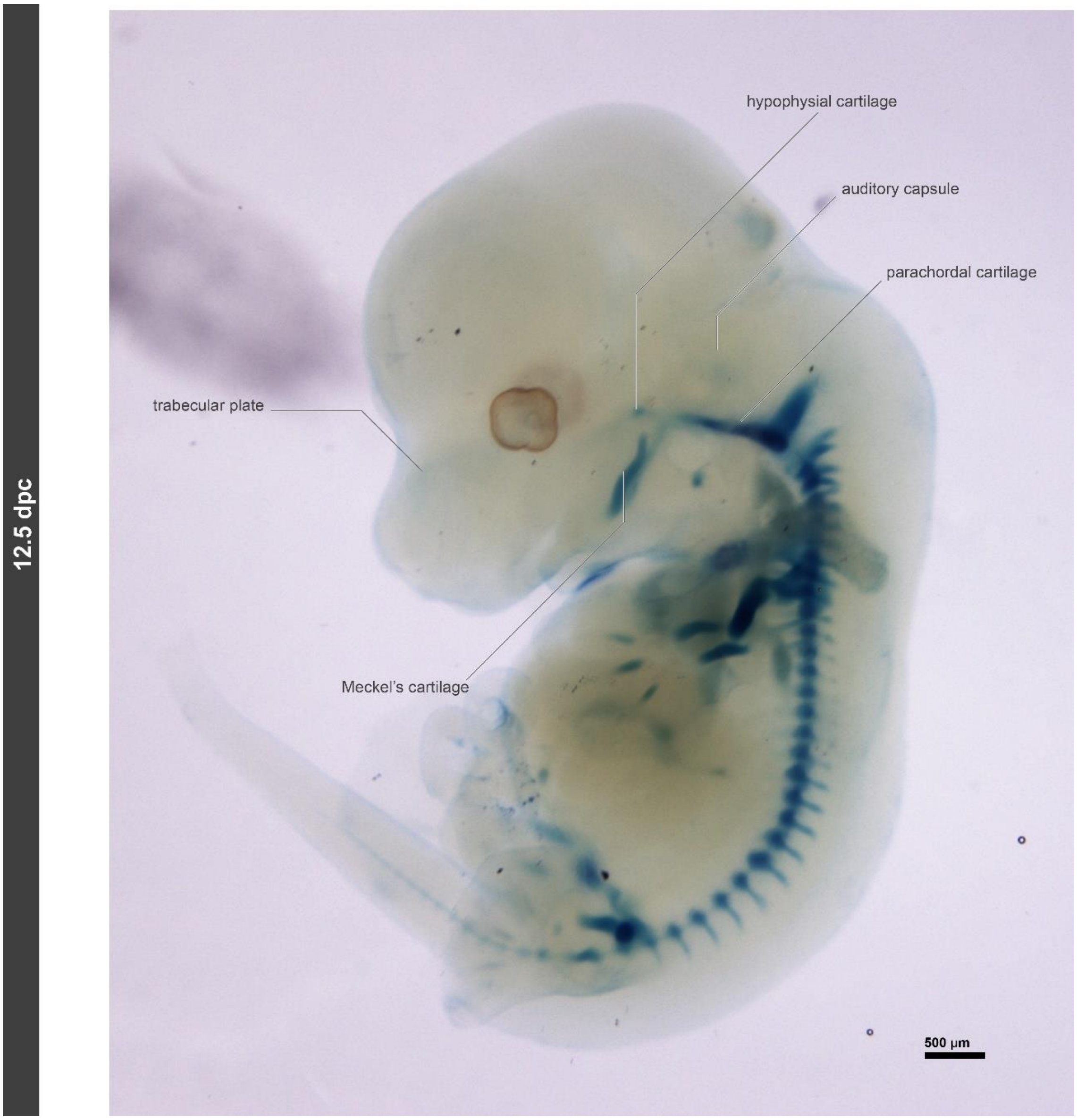

Figure S9. Alcian blue staining of 12.5 dpc mouse embryo.

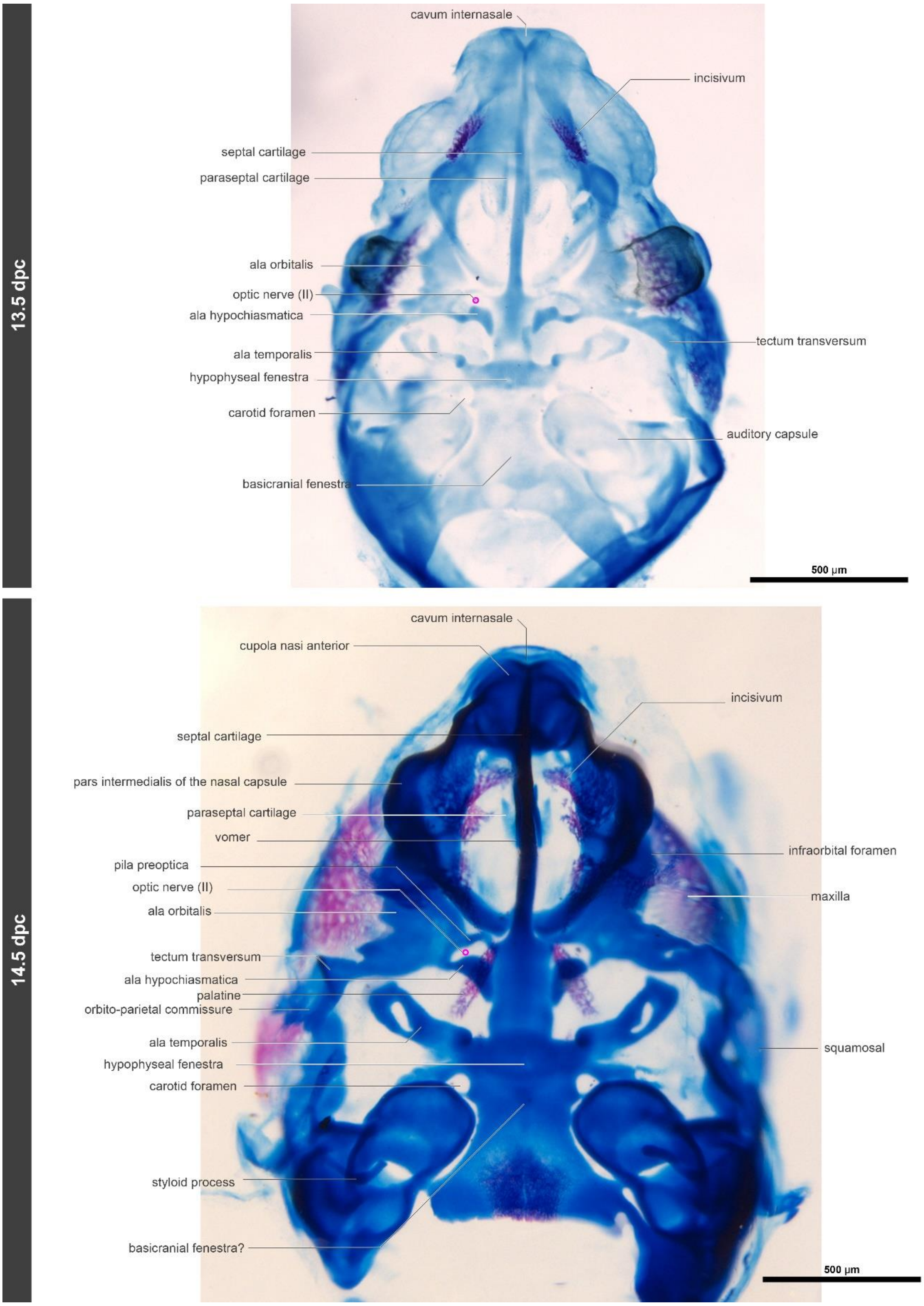

Figure S10. Arizarin red & Alcian blue staining of 13.5 and 14.5 dpc mouse embryos.
In the 14.5 dpc mouse, the basicranial fenestra is not open. It is probably a little further along in development than the individual we used for the 3D reconstruction.

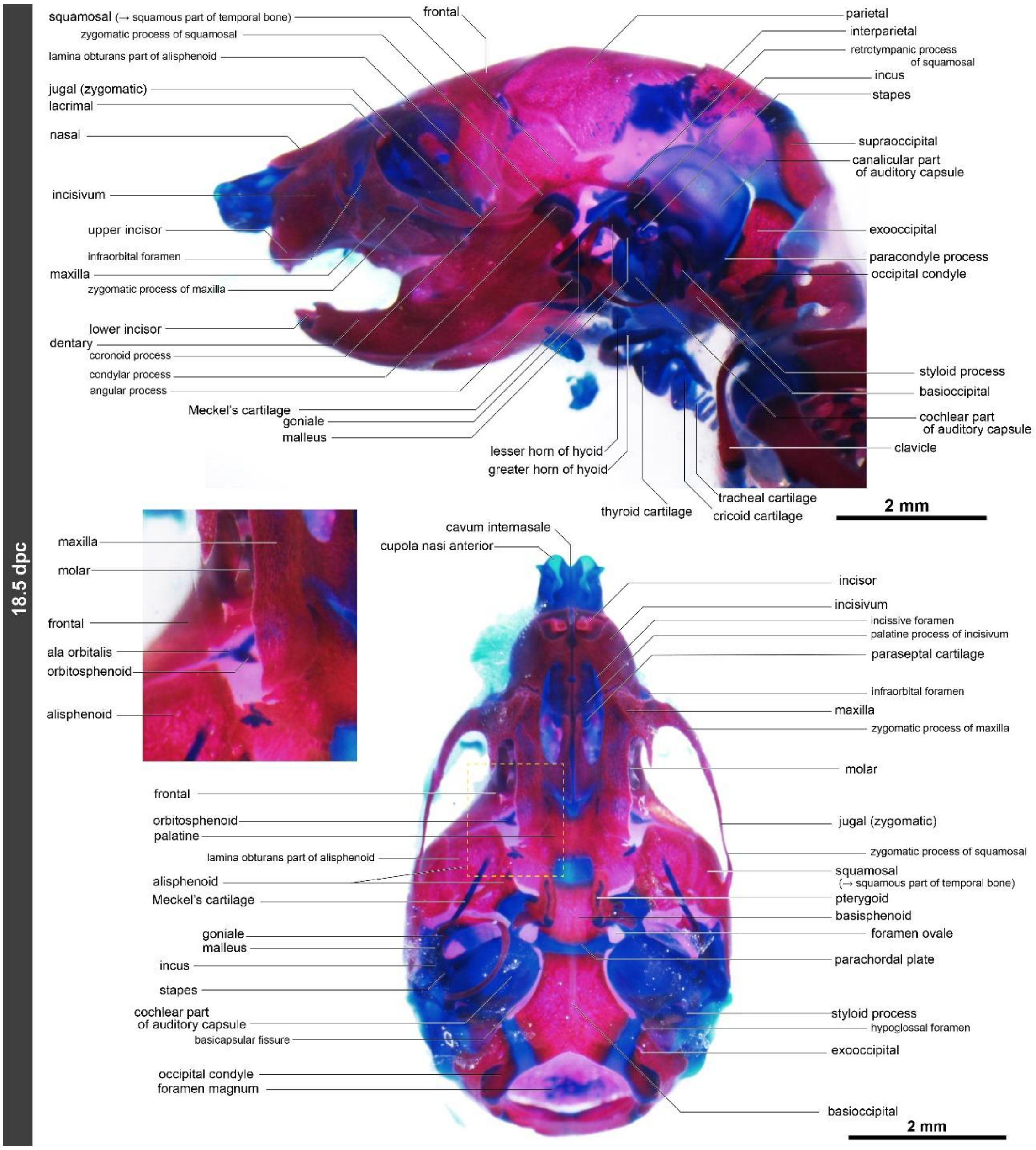

Figure S11. Arizarin red & Alcian blue staining of 18.5 dpc mouse fetus.

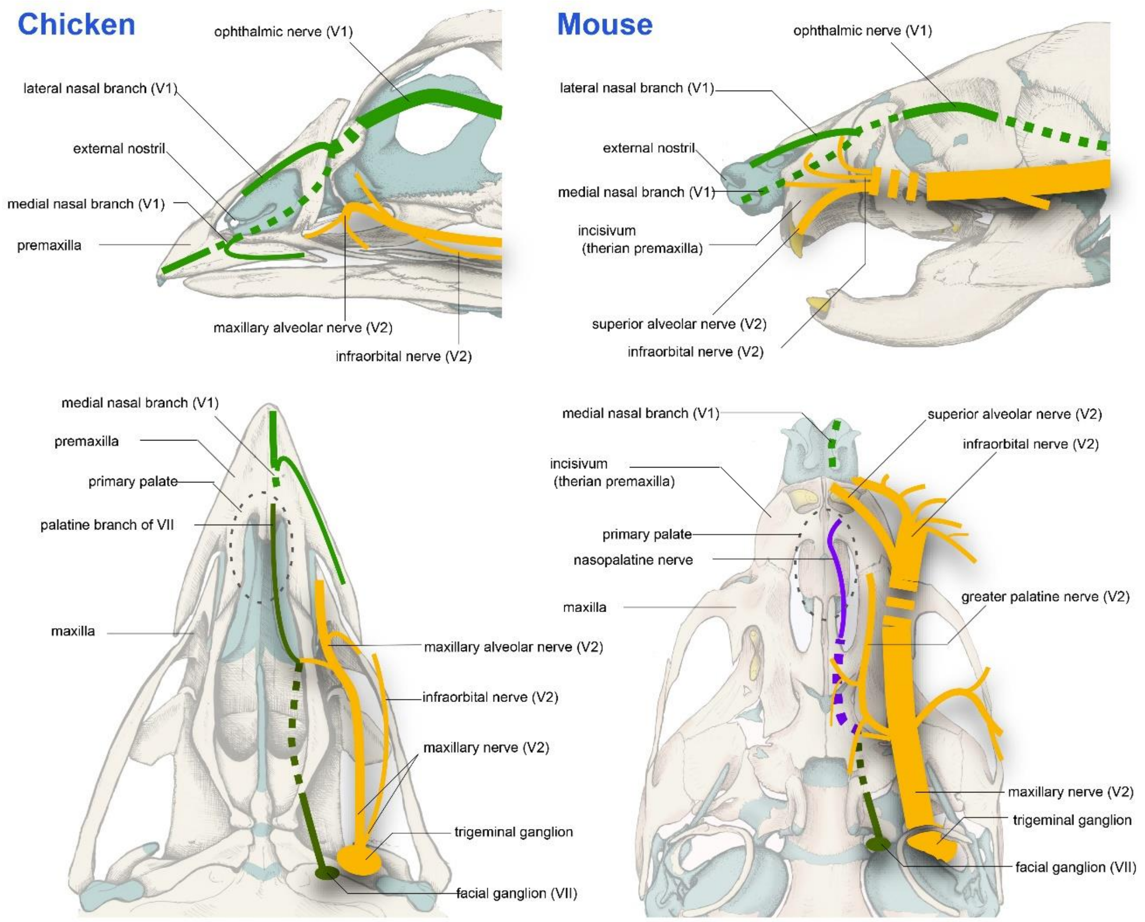

Figure S12. Maxillofacial innervation of the chicken and mouse. Reprinted with modifications from Koyabu (2023). The original drawing was created by the author of the present paper (H.H.).
